# Metal and polymer-mediated synthesis of porous crystalline hydroxyapatite nanocomposites for environmental remediation

**DOI:** 10.1098/rsos.171557

**Published:** 2018-01-24

**Authors:** Danushika C. Manatunga, Rohini M. de Silva, K. M. Nalin de Silva, Nuwan de Silva, E. V. A. Premalal

**Affiliations:** 1 Department of Chemistry, University of Colombo, Colombo 00300, Sri Lanka; 2 Sri Lanka Institute of Nanotechnology (SLINTEC), Nanotechnology and Science Park, Mahenwatte, Pitipana, Homagama 10206, Sri Lanka

**Keywords:** porous hydroxyapatite, crystallinity, environmental, alginate, metals

## Abstract

This study was focused on the preparation of metal and polymer-mediated porous crystalline hydroxyapatite (HAp) nanocomposites for environmental applications. Four different nano HAp systems were synthesized, namely, microwave irradiated HAp (M1), Zn doped HAp (M2), Mg-doped HAp (M3) and sodium alginate incorporated HAp (M4), and characterized using X-ray diffraction (XRD), Fourier transform infra-red spectroscopy, scanning electron microscopy, transmission electron microscopy, atomic force microscopy, nuclear magnetic resonance (NMR), X-ray fluorescence, thermogravimetric analysis and Brunauer–Emmett–Teller (BET) analyses. Systems M1–M4 showed morphologies similar to coral shapes, polymer-like interconnected structures, sponges and feathery mycelium assemblies. Using XRD, selected area electron diffraction patterns and ^1^H and ^31^P CP/MAS solid-state NMR studies, crystallinity variation was observed from highest to lowest in the order of M4 > M1 > M3 > M2. Surface area estimates using BET isotherm reflected the highest surface area for M3, and M1 > M2 > M4. Four systems of M1–M4 were used as potential adsorbent materials for the removal of metal containing azo dye from aqueous system. Adsorption data were correlated to Freundlich and Langmuir isotherm models. According to the results, the highest capacity of 212.8 mg g^−1^ was exhibited by M4 having mycelium like morphology with alginate groups. This study highlights the possibility of developing HAp nanocomposites for the effective removal of dye contaminants in the environment.

## 1. Introduction

Hydroxyapatite (HAp) having the chemical formula of Ca_10_(PO_4_)_6_(OH)_2_ is an inorganic biocompatible material which is known to be the most stable form of calcium phosphate [1]. The synthesis of morphologically different HAp has become one of the most interesting areas in the field of HAp chemistry, as it has a pronounced influence on properties such as mechanical strength and adsorption [1,2].

Various synthetic approaches have been reported for the synthesis of HAp nanoparticles [3], including solid-state reaction [4], sol–gel method [5,6] template-mediated synthesis [7,8], hydrothermal synthesis [7], microemulsion technique [1], co-precipitation [4–7], microwave irradiation [9] and a biomimetic method [10]. Of these methods, the co-precipitation technique has been identified as the simplest and most cost effective [2]. Size, shape, composition and crystallinity of the final product originating from co-precipitation are known to be strongly influenced by the reaction conditions [1,5], such as pH [11], temperature [2] and the ripening time [12]. During the last decade, the synthesis of micro/nanostructured HAp matrices or scaffolds, mainly using co-precipitation methods, has become much more popular, as it has made the use of HAp more widely available for many biological [13] and industrial applications [14,15]. For the purpose of developing micro/nanostructured HAp, the addition of extra materials has been reported. It has been observed that these additives can influence obtaining the desired morphology by manipulating the rate of the growth of the surface [16]. There are many reports on developing various porous morphologies using different types of additives such as chitosan [17], sodium alginate [18] and carboxymethyl cellulose [19], and in many of these studies, calcination [20] and freeze drying [17] have been introduced as additional steps. These organic–inorganic polymer composites have been generally synthesized either by co-precipitating HAp within the polymer matrix [21] or by dispersing HAp nanoparticles in the polymer solution which allows the post blending of the nanoparticles with the polymer [17]. Microwave irradiation has mainly been used to generate calcium deficient HAp [5,9]. However, many of these synthetic approaches have required lengthy preparation time [20], high temperature preparation [4], high pH medium [22], freeze drying [17], foaming agents [9] or matrices which could ultimately produce a porous body upon calcination.

Porous HAp has shown very high sorption capacities [14] and ion exchange properties [23]. Therefore, this has been used in many industrial applications, for example, catalytic supporters, sensors, and heavy metal and dye removal from water bodies [24,25,26]. Synthetic dyes are organic contaminants that are being introduced to water bodies by textile, leather, paint, paper or pharmaceutical industries [26,27]. Metal containing complex dyes are widely used in textile dyeing due to the ease of preparation and solubilization [28] and one such metal ion containing dye [29] is acid yellow 220 (AY220), which is an anionic azo dye mainly used to stain nylon, wool and silk materials [30].

It is reported that these dye molecules can severely affect the gastrointestinal tract, lungs, skin and the formation of blood [30]. Apart from the toxicity, these dye molecules are also known to increase chemical oxygen demand and reduce light penetration, making them undesirable for both flora and fauna in aquatic environments [31]. Therefore, finding a possible approach [32] for the effective removal of these dye molecules has been really challenging and many approaches have been introduced over the years [33,34]. The adsorption removal is the commonest approach and for this activated carbon has been the main choice [35]. In addition, rice husks, cotton waste, orange peel, tea waste [29,34], CaCO_3_ [36], graphene materials [37] and magnetic nanomaterials [38] have also been used.

The use of apatite materials as an alternative approach for dye removal was later recognized [39], due to its high sorption capacity [14] and ion exchange properties [33]. Nevertheless, only a few studies have been reported on the use of HAp for the removal of azo dyes [24,40]. Thus, in this study, it was attempted to synthesize efficient HAp matrices with various morphologies via different facile synthetic approaches, to study their ability to adsorb AY220, which is an anionic azo dye [29]. In order to obtain different morphologies, use of biopolymers like sodium alginate, use of microwave irradiation and metal doping were investigated. The different morphologies resulting from these approaches were analysed, and their capacity to adsorb AY220 was monitored.

## 2. Material and methods

### 2.1. Material and methods

The following chemicals were used as received without further purification. Ca(NO_3_)_2_.4H_2_O (99%), (NH_4_)_2_HPO_4_ (≥99%), MgCl_2_.6H_2_O (99–102%), Zn(NO_3_)_2_.6H_2_O (98%) and sodium alginate (low viscosity-NaAlg) were purchased from Sigma Aldrich Corporation. Ammonia solution (25%) was purchased from Merck & Co., Inc. Double-distilled water was used throughout the experiment**.**

### 2.2. Preparation of microwave-irradiated HAp scaffold (labelled as M1)

Solutions of 0.25 M Ca(NO_3_)_2_.4H_2_O and 0.16 M (NH_4_)_2_HPO_4_ were prepared using double-distilled water. The initial pH of the (NH_4_)_2_HPO_4_ and 0.25 M Ca(NO_3_)_2_.4H_2_O was adjusted to 9 and 8 respectively using ammonia solution. Then 25 ml of 0.16 M (NH_4_)_2_HPO_4_ was added dropwise to 25 ml of 0.25 M Ca(NO_3_)_2_.4H_2_O solution under vigorous stirring while maintaining the pH at 8.5–9.0. At the end of the addition, the solution was aged at the same temperature for another 2 h with vigorous stirring. The resulting white colour precipitate was washed thoroughly. Later it was subjected to microwave irradiation for 1.5 min using a domestic microwave oven operating at 2.5** **GHz, 800 W. The resulting white solid was ground and used for further characterization**.**

### 2.3. Preparation of Zn doped HAp (labelled as M2)

Solutions of 0.25 M Ca(NO_3_)_2_.4H_2_O and 0.16 M (NH_4_)_2_HPO_4_ were prepared separately. Zn(NO_3_)_2_.6H_2_O (0.25** **g) was dissolved in 25 ml of 0.25 M Ca(NO_3_)_2_ solution to prepare 40% (w/w, HAp) solution. A solution of 25 ml of 0.16 M (NH_4_)_2_HPO_4_ at pH 9 was added dropwise to this mixture under vigorous stirring. After the addition of (NH_4_)_2_HPO_4_, the reaction mixture was vigorously stirred for another 2 h while maintaining the pH at 8.5–9.0. The resulting precipitate was dried at room temperature and used for further characterization**.**

### 2.4. Preparation of Mg-doped HAp scaffold (labelled as M3)

Solutions of 0.25 M Ca(NO_3_)_2_.4H_2_O and 0.16 M (NH_4_)_2_HPO_4_ were prepared and the pH adjusted to 8–9 by adding ammonia. MgCl_2_.6H_2_O (0.25** **g) was added to 25 ml of the Ca^2+^ precursor solution and mixed thoroughly to obtain a 40% (w/w, HAp) solution. To this mixture, 25 ml of 0.16 M (NH_4_)_2_HPO_4_ was added dropwise under vigorous stirring. At the end of the addition, the stirring was continued for another 2 h, while maintaining the pH at 8.5–9.0. The resulting white gel was subjected to suction filtration and the rest of the procedure was followed as in the previous sections**.**

### 2.5. Preparation of sodium alginate/HAp scaffold (labelled as M4)

Solutions of 0.25 M Ca(NO_3_)_2_.4H_2_O and 0.16 M (NH_4_)_2_HPO_4_ were prepared. NaAlg (0.25** **g) was added to 25 ml of the (NH_4_)_2_HPO_4_ solution, and mixed thoroughly to obtain a 40% (w/w, HAp) solution, and the pH was adjusted to 9. The mixture of (NH_4_)_2_HPO_4_ and NaAlg was added to 25 ml of 0.25 M Ca(NO_3_)_2_.4H_2_O solution with vigorous stirring. At the end of the addition, the stirring was continued for another 2 h. The rest of the procedure was same as stated in the previous sections**.**

### 2.6. HAp nanoparticle characterization

#### 2.6.1. Powder X-ray diffraction studies

Powder X-ray diffraction (XRD) analysis of synthesized HAp nanoparticles was performed by recording the XRD pattern with Cu K*α* radiation (*λ* = 1.5418 Å, Bruker D8 Focus X-ray diffractometer) over the range of 5°–80°.

#### 2.6.2. Diffuse reflectance Fourier transform infrared spectroscopy

The interactions of the respective components and the formation of HAp were determined by Fourier transform infrared (FT-IR) spectra recorded using the diffuse reflectance mode over the range of 400–4000 cm^−1^ by a Bruker Vertex 80.

#### 2.6.3. Morphology characterization using scanning electron microscopy, transmission electron microscopy and atomic force microscopy

The morphology of the scaffolds was examined with scanning electron microscopy (SEM; Hitachi SU 6600). Transmission electron microscopy (TEM) investigations were performed using a JEOL 3010 at 300 kV (Indian Institute of Technology, Madras, India) and JEOL JEM 2100 at 200 kV (SLINTEC). Selected area electron diffraction (SAED) patterns were collected to observe the crystallinity of these samples. Surface topography of HAp nanoparticles was obtained using a Park System atomic force microscopy (AFM) XE-100 microscope. The measurements were taken under air at room temperature using non-contact mode with Si tips of the 1650–00 type, scanning at a rate of 0.5 Hz.

#### 2.6.4. NMR experiments

Solid-state ^1^H and ^31^P NMR (cross-polarization, magic angle spinning and dipolar decoupling (CP/MAS)) spectra were acquired at room temperature through a Bruker AVANCE-III 400 MHz spectrometer. The NMR experiments for proton and phosphorus were carried out with the spectrometer operating at 400.15 MHz and 161.98 MHz, respectively. The contact time for cross-polarization was 3.5 ms and the spin rate of the 4 mm zirconia rotor was 5 kHz. The chemical shift is reported with respect to two external standards, i.e. adamantane for ^1^H NMR and ammonium dihydrogen phosphate for ^31^P NMR.

#### 2.6.5. Elemental composition analysis by X-ray fluorescence

The chemical composition of the samples was determined by X-ray fluorescence (XRF) using an XRF microscope (Horiba XGT-5200).

#### 2.6.6. Characterization of thermal decomposition

Thermal decomposition of the respective samples was analysed using an SDT Q 600 thermogravimetric analyser where the samples were heated at a ramp of 20°C min^−1^ in air with a temperature range from room temperature to 1000°C.

#### 2.6.7. Brunauer–Emmett–Teller specific surface area determination

The specific surface area and pore size distribution of the sample powders were determined by the Brunauer–Emmett–Teller (BET) method using a Micromeritics Pulse Chemisorb 2705. The powdered samples (0.03 g) were degassed at 130.8°C for 1 h prior to analysis.

### 2.7. Removal of azo-metal textile dye

AY220, commercially known as Lanaset yellow 2R [29], was supplied by a local textile factory and was used without further purification. This dye was used as a model compound with the systems M1, M2, M3, M4 to assess their dye removal ability. The effect of pH and contact time was analysed for each system and based on those optimum values, batch adsorption studies were performed. The equilibrium data were explained by two common models, the Langmuir and Freundlich isotherms (electronic supplementary material, 1.1). The effect of contact time, effect of pH on dye removal and the details of the batch sorption experiment are given in the electronic supplementary material, 1.2–1.4**.**

Dye bound HAp nanoparticles were dried and further subjected to FT-IR spectroscopy to identify the presence of the dye molecules and data are given in the electronic supplementary material.

## 3. Results and discussion

### 3.1. Crystallographic phase characterization using X-ray diffraction

**Figure 1.**
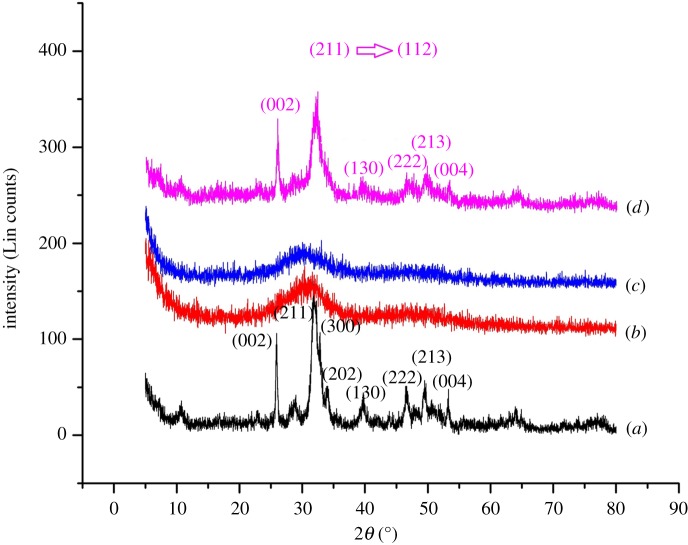
XRD pattern of each system: (*a*) M1, (*b*) M2, (*c*) M3 and (*d*) M4.

The XRD pattern of each system is displayed in figure 1, where they have been compared with the reference pattern of JCPDS.PDF.Ref.01.072.1243. The diffraction patterns, except for the pattern in M2 (figure 1*b*) and M3 (figure 1*c*), corresponded to that of hexagonal synthetic HAp and space group P63/m. In M2 and M3, the XRD spectrum is very broad and individual peaks were hard to identify.

Systems M1 and M4 have shown a well-resolved and intense peaks which could account for an increased crystallinity with respect to other systems. The M1 system, which was synthesized using microwave irradiation, is already known to produce a sudden increment of temperature [9], affecting the crystallinity [11]. Additionally, the shift of the highest intensity peak, corresponding to the phase (211) of neat HAp, to (112) in the M4 system has shown a favourable interaction of HAp particles with the added sodium alginate as previously reported [21]. The lowest crystallinity was observed with the metal-doped systems (M2 and M3). This observation is in accordance with previous reports where decreased crystallinity has been observed with metal doping [41], due to the substitution of Ca^2+^ ions by Zn^2+^ and Mg^2+^ ions [42] resulting in amorphous systems [22].

### 3.2. Fourier transform infrared spectroscopy analysis

**Figure 2.**
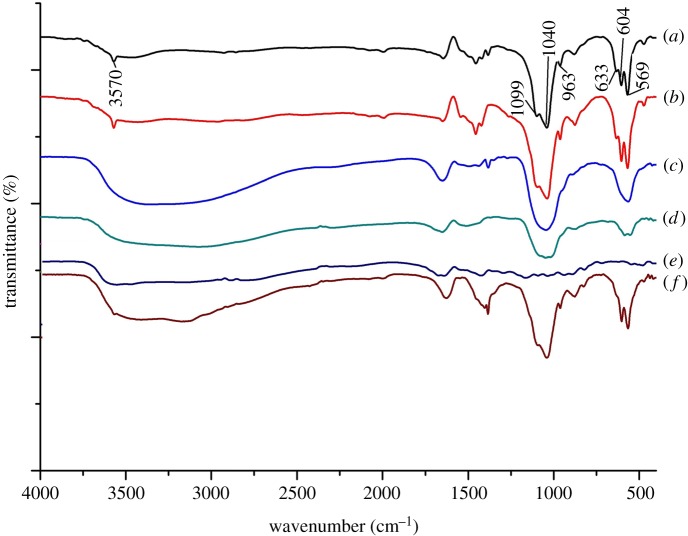
FT-IR spectra of (*a*) neat HAp, (*b*) M1, (*c*) M2, (*d*) M3, (*e*) NaAlg neat polymer and (*f*) M4.

The FT-IR spectra obtained for M1–M4, as well as neat HAp and neat polymers, are compared in figure 2. As depicted in figure 2*b*, the microwave-irradiated HAp sample (M1) is compared with neat HAp synthesized in figure 2*a*, following the same procedure in the absence of microwave irradiation. In M1, distinct bands at 3570 cm^−1^, 633 cm^−1^ and 963 cm^−1^, which correspond to the –OH stretching, –OH bending and phosphate stretching vibrations of the apatite structure, appeared [21,22]. The intensities of these two hydroxyl absorption vibrations and the band at 963 cm^−1^ of phosphate vibration can be used as an indication of the crystallinity of the HAp [16]. In addition, other phosphate vibrational bands were also observed at 569, 604, 1040, and 1099 cm^−1^ [43].

In the case where the HAp matrix has been doped with Zn^2+^ (figure 2*c*), a broad –OH stretching band appeared around 3355 cm^−1^, which could be due to the high content of adsorbed water [7,21]. This peak overlaps with the –OH stretching of apatite at 3570 cm^−1^ [6]. The higher intensity of the –OH bending peak at 1650 cm^−1^ [6,9] further supports a high amount of adsorbed water. The less crystalline nature of the metal-doped samples (M2 and M3) was evident from the single broad peak at the region of 1050 and 550 cm^−1^ as previously reported [43]. However, the shifting of the phosphate vibrational peak from 604 to 585 cm^−1^ in the Mg^2+^-doped sample (M3) in figure 2*d* can be attributed to lattice distortion [33].

When HAp was synthesized in the presence of NaAlg, the interaction of this polymer molecule with HAp was evident from the additional peaks appearing in the FT-IR spectrum of M4 as given in figure 2*e*,*f*. In neat NaAlg, the FT-IR peaks (electronic supplementary material, figure S1) at 1425 cm^−1^ and at 1036 cm^−1^ corresponded to the symmetric stretching of COO^−^ attached to the polysaccharide backbone and C–O–C vibrational bands of cyclic acetals, respectively [44]. However, in the spectrum of M4, the C–O–C vibrational band has overlapped with the phosphate stretching at approximately 1040 cm^−1^ [44]. Additionally, a small peak has appeared at 1403 cm^−1^ which could be assigned as a shift of the symmetric stretching vibration of the COO^−^ group of the NaAlg present among the HAp molecules. Also a broad –OH stretching peak has resulted from the incorporation of the polymer molecule, which has enhanced the adsorbed water content [9].

### 3.3. Morphological characterization using scanning electron microscopy, transmission electron microscopy and atomic force microscopy

**Figure 3.**
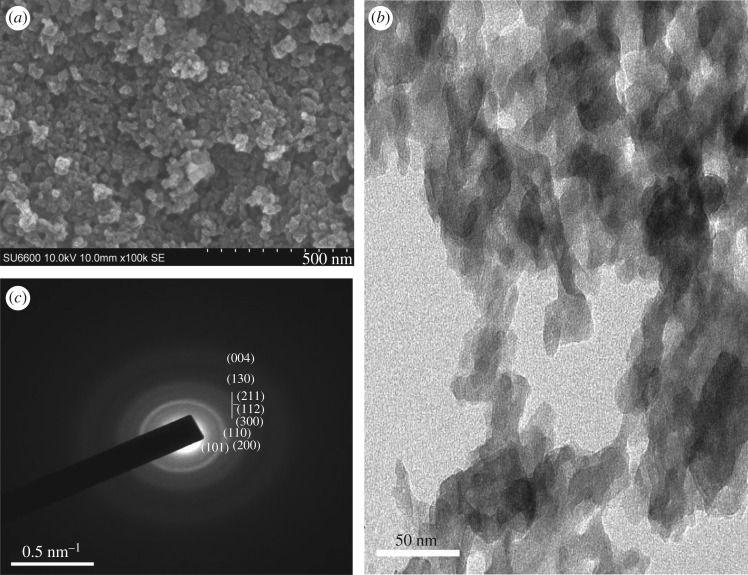
Morphology of the nanoparticles resulting from M1: (*a*) SEM image (scale bar 500** **nm), (*b*) TEM image (scale bar 50** **nm) and (*c*) SAED pattern of M1.

Morphology of the obtained HAp particles was studied using SEM, TEM and AFM. As given in figure 3*a,b*, in the M1 system, the HAp nanoparticles have interconnected in a coral-like network showing an individual particle diameter in the range of 15–20 nm. TEM pictures show particles are nearly of similar shape and size interconnected with voids confirming the presence of separated particles. During the microwave irradiation, as the sample was rapidly heated to a higher temperature [9], the nanoparticles might have arranged quickly into a scaffold like matrix without being separated as discrete nanoparticles.

The SAED pattern obtained for the M1 system (figure 3*c*) shows slightly reduced crystalline nature with the appearance of faint bands in contrast to bulk apatite [45] confirming the nano range properties. Additionally, the SAED pattern observed is in agreement with the hexagonal structure, in keeping with the XRD data.

**Figure 4.**
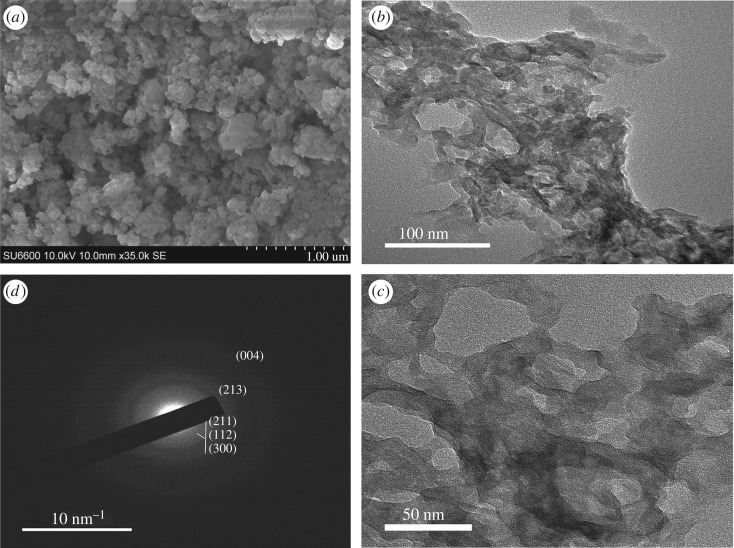
Morphology of the nanoparticles resulting from M2: (*a*) SEM image (scale bar 1000** **nm), (*b*) TEM image (scale bar 100** **nm), (*c*) TEM image at higher resolution (scale bar 50** **nm) and (*d*) SAED pattern of M2.

As depicted in figure 4*a–c*, in the M2 system where the Zn metal doping has been introduced, the resulting particles are arranged in a network creating void spaces (50–100 nm) in between. Close examination of the SEM images reveals that there are individual particles in the range of 100 nm on the surface. However, TEM images could not give any evidence of well-separated particles but show a polymer-like interconnected appearance with large voids. This type of interconnected porous structure originating from the doping of HAp with Zn^2+^ has never been reported, albeit there are few reports of Zn doping of HAp [22,43]. The SAED pattern of this system showed a very faint diffused ring pattern, proving its amorphous nature [46].

**Figure 5.**
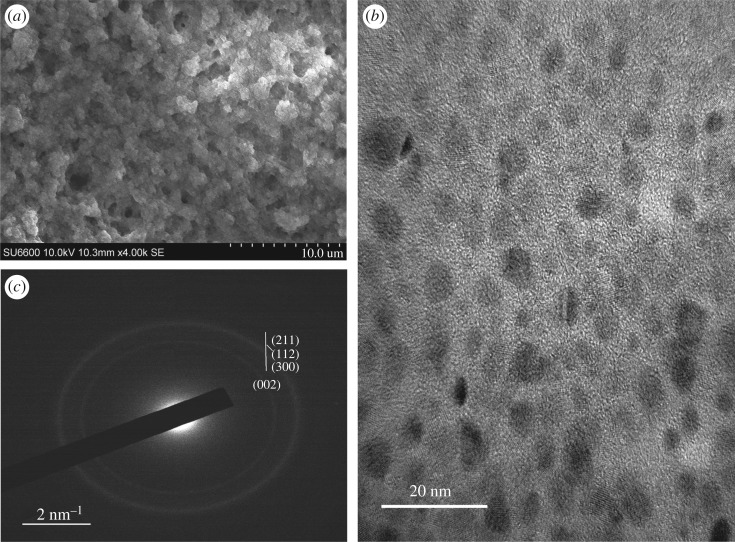
Morphology of the porous scaffold resulting from Mg doping (M3): (*a*) SEM image (scale bar 10** **µm), (*b*) TEM image of individual nanoparticles (scale bar 20** **nm) and (*c*) SAED pattern of the resulting nanoparticles.

The SEM image obtained for Mg-doped HAp (M3) shows a sponge like appearance as given in figure 5*a*, where the nanoparticles are arranged in such a way as to provide a highly porous substrate with the highest surface area of the five samples prepared, as exhibited from the BET isotherm data (electronic supplementary material, table S4). However, the TEM image (figure 5*b*) shows the existence of discrete nanoparticles in the aqueous medium, verifying that the individual particles of the coral structure are in the nano range. According to the image, the sizes of the particles are below 10 nm, with the majority of them being spherical in shape. Usually Mg-doped HAp nanoparticles exhibit morphologies of needles, rods and spheres [43,47], in contrast to this study, which showed coral-like porous structure. When the SAED pattern given in figure 5*c* is considered, a few faint rings appearing along with some spots indicate the polycrystalline and amorphous nature of the nanoparticles [48].

**Figure 6.**
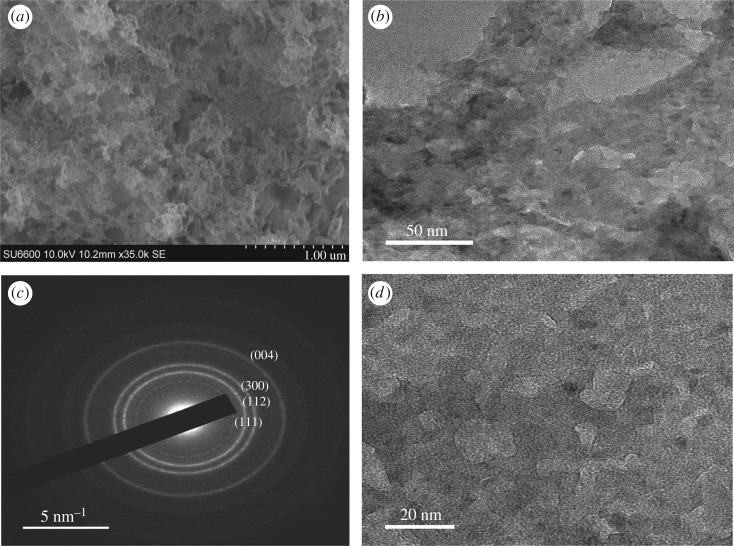
The mycelium like appearance of the M4 system: (*a*) SEM image (scale bar 1000** **nm), (*b*) TEM image of the mycelium structure (scale bar 50** **nm), (*c*) SAED pattern of the resulting nanoparticles and (*d*) TEM image of nanoparticle showing porous behaviour (scale bar 20** **nm).

The SEM image obtained for HAp synthesized in the presence of sodium alginate is given in figure 6*a*. According to the image the structure is similar to a feathery mycelium with even thickness over the entire three-dimensional network. The TEM images in figure 6*b*,*d* provide further evidence where the presence of crystal planes of HAp is clearly visible. Also from figure 6*d*, the winding arrangement of the three-dimensional polymer shows that its width is somewhat uniform, and that there are many voids creating a massive microporous structure. The formation of this kind of HAp can be easily discussed with the help of the reported ‘egg-box’ model [49].

Accordingly, addition of Ca^2+^ to the alginate polymer can mobilize Ca^2+^ ions at the centre of four monomer units of alginate polymer sites, predetermining nucleation sites for the formation of the HAp structure. As these sites are uniformly distributed within the three-dimensional network, the formation of HAp had been continuous throughout and had given a uniform thickness. The SAED pattern observed for the M4 system (figure 6*c*) exhibits well-resolved ring patterns for crystal phases, demonstrating the most crystalline nature of all systems.

The AFM images of the surface topography of HAp nanoparticles deposited on a mica sheet are given in electronic supplementary material, figure S2. The micrographs show the presence of agglomerations among the particles, which is a common characteristic irrespective of the nature of the material or the way of deposition [50,51]. Furthermore, it could be expected that the morphologies obtained from AFM images could be different from the actual ones as a result of drying and surface effects [52]. When the average particle diameter was determined along the arrow of the cross section (red and green, electronic supplementary material, figure S2*e*–*h*) it was clear that it is in accordance with the SEM and TEM observations.

### 3.4. ^1^H NMR and ^31^P NMR analysis

**Figure 7.**
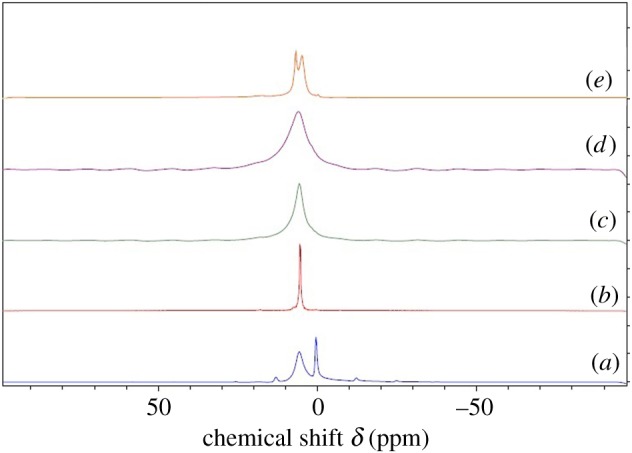
^1^H NMR spectra resulting from each system: (*a*) neat HAp, (*b*) M1, (*c*) M2, (*d*) M3 and (*e*) M4.

To determine the effect of the methodology used in the synthesis of HAp, samples were subjected to solid-state NMR analysis. As given in figure 7, the overlay of the ^1^H NMR spectra revealed two peaks for HAp, which appear around 0 and 5.3–5.5 ppm region [53]. The peak near 0–0.33 ppm accounts for the apatite –OH group of HAp [53] and it was seen in neat HAp (with high intensity) and in M1 and M4 with reduced intensity. Structural water molecules can form H-bonds among themselves or with hydroxide ions, while adsorbed water can interact with Ca^2+^ ions of HAp or form H-bonds with P--OH and P = O sites [53]. In addition, they could participate in H-bonding with the water molecules in the environment [53]. Much broader peaks for M2 and especially in M3 indicated their low crystalline amorphous nature with high water content [53]. Additionally, in M4 there was another peak appearing around 7.31 ppm highlighting tightly bound adsorbed water as previously reported [54]. The line width of the peak corresponding to surface adsorbed water of each sample was compared and tabulated (electronic supplementary material, table S1), and it was found that the sample M3 was the one with highest amount of water.

**Figure 8.**
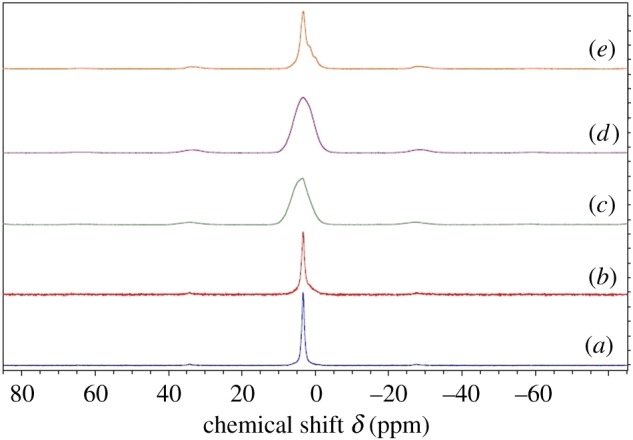
^31^P NMR spectra resulting from each system: (*a*) neat HAp, (*b*) M1, (*c*) M2, (*d*) M3 and (*e*) M4.

As given in figure 8, ^31^P NMR spectra obtained for these systems show a peak around 3.1 ppm, characteristic of apatite structure [53]. This peak in M4 has shifted to a lower frequency (electronic supplementary material, table S2) when compared with the peak value of neat HAp. This could be due to the increase of magnetic shielding of phosphorus nuclei, which could result from the higher electron density of the P atoms. On the other hand, the same peak in M2 and M3 has shifted to a higher frequency region, which highlights the magnetic de-shielding of the ^31^P nuclei in these systems [55]. Much broader peaks observed for M2 and M3 further highlighted the lower degree of crystallinity associated with these samples. The additional ‘foot’ in the ^31^P NMR of the system M1 indicated the presence of nano range particles, and it was difficult to examine this foot feature in other systems due to the broad nature of the peak [56].

### 3.5. X-ray fluorescence studies

According to the Ca/P ratio obtained for all samples by the XRF analysis, almost all of the systems were low crystalline calcium deficient HAp compared with pure HAp (electronic supplementary material, table S3). In M1, the Ca/P ratio was between 0.8 and 1.3, which highlights the coexistence of octacalcium phosphate with HAp, in accordance with the work reported by Guha *et al*. [57]. The lowest Ca/P ratio was observed with M2 and M3 samples, which exhibited the formation of amorphous calcium phosphate (ACP) in keeping with experimental results obtained from XRD, SAED patterns, and ^1^H and ^31^P NMR. ACP is an intermediate phase during the preparation of calcium phosphate via the precipitation technique [58], and it can be stabilized by adding inorganic ions like Mg^2+^ and Zn^2+^ [14,58]. Additionally, these cations are also known to act as inhibitors for the conversion of ACP to HAp [58] supporting the observed low crystallinity of M2 and M3 samples.

The system M4 led to a Ca/P ratio of 1.57, with high crystallinity, closer to pure HAp, and this was clearly supported by the SAED pattern (figure 6*c*), XRD, and ^31^P NMR and ^1^H NMR. The formation of highly crystalline M4 is in accordance with the previously published data [21].

### 3.6. Thermal decomposition of the HAp nanoparticles

**Figure 9.**
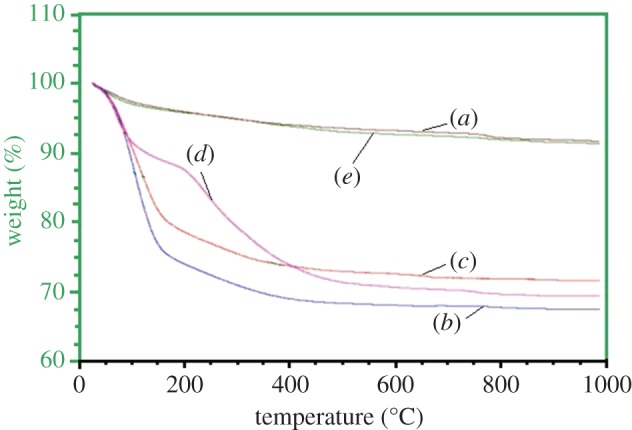
Thermal decomposition of (*a*) M1, (*b*) M2, (*c*) M3 and (*d*) M4 compared with a standard neat HAp sample (*e*).

As depicted in figure 9, the thermal decomposition of M1– M4 systems, together with neat HAp, showed an initial weight loss in the region of 30–200°C, due to the removal of adsorbed water [59]. There is a huge weight loss in the region of 30–150°C for M2 and M3 samples, supporting their amorphous nature and greater ability to bind a large amount of water, as supported by ^1^H NMR. The second weight loss seen in the region 200–400°C is due to the removal of lattice bound water [53]. Additionally, this weight loss has also been attributed to the conversion of $\text{HP}{{\text{O}}_{4}}^{2-}$ to pyrophosphate $\left({\text{P}}_{2}{{\text{O}}_{7}}^{4-}\right)$ in amorphous HAp samples [6]. The third loss observed for the samples in the region 400–800°C is due to the elimination of carbonate ions bound to HAp [56]. In M4, an additional weight reduction observed after 400°C demonstrated the decomposition of alginate polymer [60].

### 3.7. BET surface area characterization of HAp nanoparticles

The BET isotherm plots obtained for each system are given in electronic supplementary material, figure S3, and exhibited different shapes in their adsorption and desorption hysteresis loops, which explains the difference of the pore structure of these systems (M1–M4). According to the International Union of Pure and Applied Chemistry (IUPAC), systems M1 and M3 can be classified as type IV isotherms giving rise to an H1 hysteresis loop [61,62]. When considering their pore sizes (electronic supplementary material, table S4), M3 exhibited the narrowest pore size having a diameter of 6.78 nm while a value of 10.55 nm pore size for the M1 system revealing typical mesoporous behaviour [62,63]. Systems M2 and M4 corresponded to a type IV isotherm with an H3 hysteresis loop [64]. Furthermore, the pore volume and the surface area calculated for each system are tabulated in the electronic supplementary material, table S4. The M3 system has the highest surface area. Relatively large specific area, pore volume and the mesoporous/nanoporous structure of the synthesized M1–M4 HAp systems highlighted their potential application as adsorbent materials.

### 3.8. Acid yellow 220 dye removal and isotherm studies

The ability of the synthesized M1–M4 HAp systems to remove organic dyes dissolved in water was studied using AY220 dye as the model. The optimal contact time for the adsorption of the acid dye was measured for all the systems. As given in electronic supplementary material, figure S4 and table S5, the fastest removal was observed with the metal-doped systems M2 and M3, which reached equilibrium after 5 min of incubation, where the adsorption capacities were 53.9 mg g^−1^ (26.93% removal) and 60.1 mg g^−1^ (32.49% removal) respectively compared to 68.6 mg g^−1^ (65.72% removal) displayed by M4 system which required a higher contact time of about 1 h. System M1 demonstrated low removal percentage (24.97%) with higher contact times. Nevertheless, in all systems, the dye adsorption is rapid within the first 3 min and later the rate becomes much slower, due to reduction of the available number of active sites with time [29].

The effect of pH on removing acid yellow dye was studied by varying the pH from 2.5 to 7.5 and results are given in the electronic supplementary material, figure S5. According to the results, the sorption capacities are higher for all the systems at acidic pH rather than alkaline. This could be because the increase of pH leads to a higher content of OH^−^ ions which compete with the dye molecules to get adsorbed to the same active sites on HAp systems, resulting in reduced adsorption capacity [29]. Therefore, the optimal pH range could be identified as 2.5–4.5.

After finding the optimum pH and the contact time for the optimal adsorption, the effect of initial dye concentration was assessed by varying the amount of the dye at a constant weight of the adsorbent. An attempt was made to fit the data obtained to the two isotherm models, Freundlich and Langmuir, as given in the electronic supplementary material, figures S6 and S7. Even though the optimum pH was around 2.5–4.5, the pH of the experimental medium was maintained at 5–6 during the study, as the maintenance of the low pH is not practically possible in industrial applications. Table 1 summarizes the respective values obtained from each isotherm model for the adsorption of acid yellow dye by M1–M4 systems.

**Table 1.** Summary of the isotherm adsorption parameters for AY220 removal by M1, M2, M3 and M4.

model		M1	M2	M3	M4
Langmuir	*b* (l m^−1^)	0.0034	(−)	0.0412	0.0004
			0.0019		
	*q*_m_ (mg g^−1^)	169.5	(−)	103.1	212.8
			312.5		
	*R*^2^	0.8655	0.4353	0.9470	0.9902
Freundlich	*K*_f_	1.02	0.33	8.70	0.12
	*n*	1.25	0.84	2.18	1.36
	*R*^2^	0.9648	0.9813	0.8094	0.9937

According to the results only the M4 system correlates with both isotherm models, while M3 correlates with the Langmuir model, and M1 and M2 correlate better with the Freundlich model. The adsorption data revealed that the dye adsorption capacity of M4 was the highest among the systems approaching a value of 212.8 mg g^−1^. The *n* value being greater than one in M1, M3 and M4 suggests that the adsorption of dye molecules to HAp nanoparticles in these systems is a favourable process; the appearance of new adsorption sites are suggested to increase adsorption capacity [42]. The negative slope observed with low *R*^2^-value explains the non-correlation of M2 with Langmuir model. This type of observation has been reported with other systems, where it has been suggested that such systems do not follow the assumptions of the Langmuir model [65,66]. However, the higher *R*^2^-value of M2 for the Freundlich model suggests that it can be explained well using the latter.

As the systems M1 and M2 fit well with the Freundlich isotherm model, it can be deduced that the adsorption of the dye could occur via a multilayer sorption process [40] which is more a physical adsorption process [66]. In the system M3, which fits well with the Langmuir model, it can be assumed that the surface of the adsorbent is homogeneous in nature [15]. However, when comparing all the systems, it is clear that the system M4 has extraordinary ability to adsorb acid yellow dye molecules, and the mechanism of adsorption could be explained well with both Langmuir and Freundlich models, owing to high *R*^2^-value. The presence of carboxylate groups [21] in its structure could be a reason for M4 to possess a higher affinity for dye molecules. However, the *n* value being higher than one in this system suggests that the adsorption of the dye molecules to M4 is more a physisorption than a chemisorption process [66]. Moreover, this higher sorption capacity resulting for M4 system is comparable with the previous work carried out by our groups which used chitosan--HAp and carboxymethylcellulose--HAp composites for dye removal [67]. It further suggests that the presence of polymer molecules further enhances the dye binding ability of these HAp nanoparticles.

Nevertheless, the sorption capacities obtained for systems M1, M2, M3 and M4 in this study are much higher than those of most commonly used adsorbents for the removal of AY220 [29,68] highlighting the superiority as novel superadsorbent materials for the rapid removal of AY220. These materials are comparable to newly reported products [69–72] and may prove to be good sorbents for other dyes as well. In addition, these adsorbent materials are associated with additional advantages such as the facile synthesis and low cost avoiding the use of templates and calcination to obtain porous matrices.

The binding of the dye to the HAp nanoparticles was further confirmed by careful observation of dye bound HAp nanoparticles, and FT-IR spectra for the samples after adsorption which are given in the electronic supplementary material, figures S8 and S9

## 4. Conclusion

This study synthesizes HAp nanoparticles via different facile synthetic approaches, and investigates their use as adsorbent materials for the removal of the azo dye AY220. The results of XRD, solid-state NMR, SEM and TEM analyses confirmed the formation of nanoparticles with varying crystallinity and shape, depending on the approach that has been employed. In most of these systems, the removal of the dye was rapid and the contact time was very low for M2 and M3 (3–5 min). As far as the microstructures of HAp systems are concerned, higher crystallinity, smaller pore size and lower surface area were observed for M1 and M4 systems and, in general, these systems exhibited a higher adsorption capacity of 169.5 and 212.8 mg g^−1^ (M1 and M4) with respect to M2 and M3. Specifically, with system M1, the organization of the nanoparticles into a coral-like shape together with the nanoporous behaviour might have triggered its dye adsorption capacity. Similarly, the system M4, which is also somewhat crystalline like M1, has the highest adsorption capacity which might have resulted from its feathery mycelium like appearance, nanoporous behaviour and the presence of chelating groups such as carboxylates in the alginate blend.

When it comes to amorphous structures seen in M2 and M3 systems, the importance of having smaller pore size together with higher surface area aiding the adsorption was revealed, with the M3 system having a pore size of 6.78 nm and highest surface area of 126.5 m^2^ g^−1^ compared to the M2 system. Further the presence of a polymer-like interconnected hollow structure in M3 might have further strengthened this phenomenon. However, in general, the effect of having polymer ligands containing binding sites may compensate for effects originating from surface area, as indicated by the M4 system. Therefore, it appears that one of the major factors in controlling the adsorption was the ligand attached to the HAp microstructure irrespective of the pore size and morphology for the crystalline systems. However, in the absence of such a driving force in amorphous systems such as M3, pore sizes, hollow architecture and surface area may play a major role.

## Supplementary Material

Characterization data and Isotherm data

## Data accessibility

This article has no additional data.

## References

[1] Paz A, Guadarrama D, López M, González JE, Brizuela N, Aragón J 2012 A comparative study of hydroxyapatite nanoparticles synthesized by different routes. Quim. Nova 35, 1724–1727. (doi:10.1590/S0100-40422012000900004)

[2] Wang P, Li C, Gong H, Jiang X, Wang H, Li K 2010 Effects of synthesis conditions on the morphology of hydroxyapatite nanoparticles produced by wet chemical process. Powder Technol. 203, 315–321. (doi:10.1016/j.powtec.2010.05.023)

[3] Monmaturapoj N 2008 Nano-size hydroxyapatite powders preparation by wet-chemical precipitation route. J. Met. Mater. Miner. 18, 15–20.

[4] Okada M, Matsumoto T 2015 Synthesis and modification of apatite nanoparticles for use in dental and medical applications. Jpn. Dent. Sci. Rev. 51, 85–95. (doi:10.1016/j.jdsr.2015.03.004)

[5] Ferraz MP, Monteiro FJ, Manuel CM 2004 Hydroxyapatite nanoparticles: a review of. J. Appl. Biomater. 2, 74–80. (doi:1722-6899/074-07$15.00/0) 20803440

[6] Shu C, Xianzhu Y, Zhangyin X, Guohua X, Hong L, Kangde Y 2007 Synthesis and sintering of nanocrystalline hydroxyapatite powders by gelatin-based precipitation method. Ceram. Int. 33, 193–196. (doi:10.1016/j.ceramint.2005.09.001)

[7] Martins MA, Santos C, Almeida MM, Costa MEV 2008 Hydroxyapatite micro- and nanoparticles: nucleation and growth mechanisms in the presence of citrate species. J. Colloid Interface Sci. 318, 210–216. (doi:10.1016/j.jcis.2007.10.008) 17996882 10.1016/j.jcis.2007.10.008

[8] Monreal Romero HA, Mora Ruacho J, Martínez Pérez CA, García Casillas PE 2013 Synthesis of hydroxyapatite nanoparticles in presence of a linear polysaccharide. J. Mater. 2013, 683268 (doi:10.1155/2013/683268)

[9] Li B, Chen X, Guo B, Wang X, Fan H, Zhang X 2009 Fabrication and cellular biocompatibility of porous carbonated biphasic calcium phosphate ceramics with a nanostructure. Acta Biomater. 5, 134–143. (doi:10.1016/j.actbio.2008.07.035) 18799376 10.1016/j.actbio.2008.07.035

[10] Sadjadi MAS, Meskinfam M, Sadeghi B, Jazdarreh H, Zare K 2011 *In situ* biomimetic synthesis and characterization of nano hydroxyapatite in gelatin matrix. J. Biomed. Nanotechnol. 7, 450–454. (doi:10.1166/jbn.2011.1305) 21830488 10.1166/jbn.2011.1305

[11] Viswanath B, Ravishankar N 2008 Controlled synthesis of plate-shaped hydroxyapatite and implications for the morphology of the apatite phase in bone. Biomaterials 29, 4855–4863. (doi:10.1016/j.biomaterials.2008.09.001) 18834629 10.1016/j.biomaterials.2008.09.001

[12] Fujii S, Okada M, Furuzono T 2007 Hydroxyapatite nanoparticles as stimulus-responsive particulate emulsifiers and building block for porous materials. J. Colloid Interface Sci. 315, 287–296. (doi:10.1016/j.jcis.2007.06.071) 17681523 10.1016/j.jcis.2007.06.071

[13] Reinares-Fisac D, Veintemillas-Verdaguer S, Fernández-Díaz L 2017 Conversion of biogenic aragonite into hydroxyapatite scaffolds in boiling solutions. CrystEngComm 19, 110–116. (doi:10.1039/C6CE01725H)

[14] Salah TA, Mohammad AM, Hassan MA, El-Anadouli BE 2014 Development of nano-hydroxyapatite/chitosan composite for cadmium ions removal in wastewater treatment. J. Taiwan Inst. Chem. Eng. 45, 1571–1577. (doi:10.1016/j.jtice.2013.10.008)

[15] Mousa SM, Ammar NS, Ibrahim HA 2016 Removal of lead ions using hydroxyapatite nano-material prepared from phosphogypsum waste. J. Saudi Chem. Soc. 20, 357–365. (doi:10.1016/j.jscs.2014.12.006)

[16] Pang YX, Bao X 2003 Influence of temperature, ripening time and calcination on the morphology and crystallinity of hydroxyapatite nanoparticles. J. Eur. Ceram. Soc. 23, 1697–1704. (doi:10.1016/S0955-2219(02)00413-2)

[17] Ramli RA, Adnan R, Bakar MA, Masudi SM 2011 Synthesis and characterisation of pure nanoporous hydroxyapatite. J. Phys. Sci. 22, 25–37.

[18] Sun J, Tan H 2013 Alginate-based biomaterials for regenerative medicine applications. Materials 6, 1285–1309. (doi:10.3390/ma6041285) 28809210 10.3390/ma6041285 PMC5452316

[19] Jiang L, Li Y, Wang X, Zhang L, Wen J, Gong M 2008 Preparation and properties of nano-hydroxyapatite/chitosan/carboxymethyl cellulose composite scaffold. Carbohydr. Polym. 74, 680–684. (doi:10.1016/j.carbpol.2008.04.035)

[20] Nathanael AJ, Lee JH, Mangalaraj D, Hong SI, Oh TH 2014 Influence of processing method on the properties of hydroxyapatite nanoparticles in the presence of different citrate ion concentrations. Adv. Powder Technol. 25, 551–559. (doi:10.1016/j.apt.2013.09.005)

[21] Rajkumar M, Meenakshisundaram N, Rajendran V 2011 Development of nanocomposites based on hydroxyapatite/sodium alginate: synthesis and characterisation. Mater. Charact. 62, 469–479. (doi:10.1016/j.matchar.2011.02.008)

[22] Devanand Venkatasubbu G, Ramasamy S, Ramakrishnan V, Kumar J 2011 Nanocrystalline hydroxyapatite and zinc-doped hydroxyapatite as carrier material for controlled delivery of ciprofloxacin. 3 Biotech 1, 173–186. (doi:10.1007/s13205-011-0021-9) 10.1007/s13205-011-0021-9 PMC3339602 22611528

[23] Park S, Gomez-flores A, Chung YS, Kim H 2015 Removal of cadmium and lead from aqueous solution by hydroxyapatite / chitosan hybrid fibrous sorbent: kinetics and equilibrium studies. J. Chem. 2015, 396290 (doi:10.1155/2015/396290)

[24] Ciobanu G, Harja M, Rusu L, Mocanu AM, Luca C 2014 Acid black 172 dye adsorption from aqueous solution by hydroxyapatite as low-cost adsorbent. Korean J. Chem. Eng. 31, 1021–1027. (doi:10.1007/s11814-014-0040-4)

[25] Adeogun AI, Babu RB 2015 One-step synthesized calcium phosphate-based material for the removal of alizarin S dye from aqueous solutions: isothermal, kinetics, and thermodynamics studies. Appl. Nanosci. 5, 1–32. (doi:10.1007/s13204-015-0484-9)

[26] Barka N, Qourzal S, Assabbane A, Nounah A, Ait-Ichou Y 2011 Removal of reactive yellow 84 from aqueous solutions by adsorption onto hydroxyapatite. J. Saudi Chem. Soc. 15, 263–267. (doi:10.1016/j.jscs.2010.10.002)

[27] Feng B, Xu X, Xu W, Zhou G, Hu J, Wang Y, Bao Z 2015 Self-assembled 3D ACF-rGO-TiO_2_ composite as efficient and recyclable spongy adsorbent for organic dye removal. Mater. Des. 83, 522–527. (doi:10.1016/j.matdes.2015.06.061)

[28] Lemlikchi W, Sharrock P, Fiallo M, Nzihou A, Mecherri MO 2014 Hydroxyapatite and alizarin sulfonate ARS modeling interactions for textile dyes removal from wastewaters. Procedia Eng. 83, 378–385. (doi:10.1016/j.proeng.2014.09.032)

[29] Deniz F, Karaman S 2011 Removal of an azo-metal complex textile dye from colored aqueous solutions using an agro-residue. Microchem. J. 99, 296–302. (doi:10.1016/j.microc.2011.05.021)

[30] Ashraf MA, Hussain M, Mahmood K, Wajid A, Yusof M, Alias Y, Yusoff I 2013 Removal of acid yellow-17 dye from aqueous solution using eco-friendly biosorbent. Desalin. Water Treat. 51, 4530–4545. (doi:10.1080/19443994.2012.747187)

[31] Pajootan E, Arami M, Mahmoodi NM 2012 Binary system dye removal by electrocoagulation from synthetic and real colored wastewaters. J. Taiwan Inst. Chem. Eng. 43, 282–290. (doi:10.1016/j.jtice.2011.10.014)

[32] Ladhe UV, Patil PR 2014 Removal of yellow 2G dye from aqueous solutions using activated carbon prepared from mosambi and cotton an agricultural waste. IOSR J. Environ. Sci. Toxicol. Food Technol. 8, 49–54. (doi:10.9790/2402-08164954)

[33] El Haddad M, Mamouni R, Saffaj N, Lazar S 2012 Removal of a cationic dye---basic red 12---from aqueous solution by adsorption onto animal bone meal. J. Assoc. Arab Univ. Basic Appl. Sci. 12, 48–54. (doi:10.1016/j.jaubas.2012.04.003)

[34] Dehghani MH, Mahdavi P 2014 Removal of acid 4092 dye from aqueous solution by zinc oxide nanoparticles and ultraviolet irradiation. Desalin. Water Treat. 54, 3464–3469. (doi:10.1080/19443994.2014.913267)

[35] Demirbas A 2009 Agricultural based activated carbons for the removal of dyes from aqueous solutions: a review. J. Hazard. Mater. 167, 1–9. (doi:10.1016/j.jhazmat.2008.12.114) 19181447 10.1016/j.jhazmat.2008.12.114

[36] Zhao M, Chen Z, Lv X, Zhou K, Zhang J, Tian X, Ren X, Mei X 2017 Preparation of core–shell structured CaCO_3_ microspheres as rapid and recyclable adsorbent for anionic dyes. R. Soc. open Sci. 4, 170697 (doi:10.1098/rsos.170697) 28989771 10.1098/rsos.170697 PMC5627111

[37] Zhao C, Guo J, Yang Q, Tong L, Zhang J, Zhang J, Gong C, Zhou J, Zhang Z 2015 Preparation of magnetic Ni@graphene nanocomposites and efficient removal organic dye under assistance of ultrasound. Appl. Surf. Sci. 357, 22–30. (doi:10.1016/j.apsusc.2015.08.031)

[38] Hozhabr Araghi S, Entezari MH 2015 Amino-functionalized silica magnetite nanoparticles for the simultaneous removal of pollutants from aqueous solution. Appl. Surf. Sci. 333, 68–77. (doi:10.1016/j.apsusc.2015.01.211)

[39] El Boujaady H, El Rhilassi A, Bennani-Ziatni M, El Hamri R, Taitai A, Lacout JL 2011 Removal of a textile dye by adsorption on synthetic calcium phosphates. Desalination 275, 10–16. (doi:10.1016/j.desal.2011.03.036)

[40] Saber-Samandari S, Saber-Samandari S, Heydaripour S, Abdouss M 2016 Novel carboxymethyl cellulose based nanocomposite membrane: synthesis, characterization and application in water treatment. J. Environ. Manage. 166, 457–465. (doi:10.1016/j.jenvman.2015.10.045) 26560638 10.1016/j.jenvman.2015.10.045

[41] Lijuan X, Liuyun J, Chengdong X, Lixin J 2014 Effect of different synthesis conditions on the microstructure, crystallinity and solubility of Mg-substituted hydroxyapatite nanopowder. Adv. Powder Technol. 25, 1142–1146. (doi:10.1016/j.apt.2014.02.019)

[42] Allam K, El Bouari A, Belhorma B, Bih L 2016 Removal of methylene blue from water using hydroxyapatite submitted to microwave irradiation. J. Water Resour. Prot. 8, 358–371. (doi:10.4236/jwarp.2016.83030)

[43] Dasgupta S, Banerjee SS, Bandyopadhyay A, Bose S 2010 Zn- and Mg-doped hydroxyapatite nanoparticles for controlled release of protein. Langmuir 26, 4958–4964. (doi:10.1021/la903617e) 20131882 10.1021/la903617e PMC2862579

[44] Zhang J, Wang Q, Wang A 2010 In situ generation of sodium alginate/hydroxyapatite nanocomposite beads as drug-controlled release matrices. Acta Biomater. 6, 445–454. (doi:10.1016/j.actbio.2009.07.001) 19596091 10.1016/j.actbio.2009.07.001

[45] Sasaki Y, Yamane S, Kurosu K, Sawada SI, Akiyoshi K 2012 Templated formation of hydroxyapatite nanoparticles from self-assembled nanogels containing tricarboxylate groups. Polymers 4, 1056–1064. (doi:10.3390/polym4021056)

[46] Mahamid J, Sharir A, Addadi L, Weiner S 2008 Amorphous calcium phosphate is a major component of the forming fin bones of zebrafish: indications for an amorphous precursor phase. Proc. Natl Acad. Sci. USA 105, 12 748–12 753. (doi:10.1073/pnas.0803354105) 10.1073/pnas.0803354105 PMC2529085 18753619

[47] Gozalian A, Behnamghader A, Daliri M, Moshkforoush A 2011 Synthesis and thermal behavior of Mg-doped calcium phosphate nanopowders via the sol gel method. Sci. Iran. 18, 1614–1622. (doi:10.1016/j.scient.2011.11.014)

[48] Cacciotti I, Bianco A, Lombardi M, Montanaro L 2009 Mg-substituted hydroxyapatite nanopowders: synthesis, thermal stability and sintering behaviour. J. Eur. Ceram. Soc. 29, 2969–2978. (doi:10.1016/j.jeurceramsoc.2009.04.038)

[49] Kühbeck D, Mayr J, Häring M, Hofmann M, Quignard F, Díaz Díaz D 2015 Evaluation of the nitroaldol reaction in the presence of metal ion-crosslinked alginates. New J. Chem. 39, 2306–2315. (doi:10.1039/C4NJ02178A)

[50] Mir M, Leite FL, Herrmann Junior PS de P, Pissetti FL, Rossi AM, Moreira EL, Mascarenhas YP 2012 XRD, AFM, IR and TGA study of nanostructured hydroxyapatite. Mater. Res. 15, 622–627. (doi:10.1590/S1516-14392012005000069)

[51] Nikpour MR, Rabiee SM, Jahanshahi M 2012 Synthesis and characterization of hydroxyapatite/chitosan nanocomposite materials for medical engineering applications. Compos. Part B Eng. 43, 1881–1886. (doi:10.1016/j.compositesb.2012.01.056)

[52] Mocanu A, Pasca RD, Tomoaia G, Garbo C, Frangopol PT, Horovitz O, Tomoaia-Cotisel M 2013 New procedure to synthesize silver nanoparticles and their interaction with local anesthetics. Int. J. Nanomedicine 8, 3867–3874. (doi:10.2147/IJN.S51063) 24143090 10.2147/IJN.S51063 PMC3797620

[53] Pajchel L, Kolodziejski W 2013 Solid-state MAS NMR, TEM, and TGA studies of structural hydroxyl groups and water in nanocrystalline apatites prepared by dry milling. J. Nanoparticle Res. 15, 1–15. (doi:10.1007/s11051-013-1868-y) 10.1007/s11051-013-1868-y PMC3751289 23990754

[54] Schmidt-rohr K, Hong M 2007 NMR investigations of biological and synthetic phosphate-based nanocomposites. PhD thesis, Iowa State University, IA, USA.

[55] Kurgan N, Karbivskyy V, Kasyanenko V 2015 Morphology and electronic structure of nanoscale powders of calcium hydroxyapatite. Nanoscale Res. Lett. 10, 41 (doi:10.1186/s11671-015-0770-1) 25852338 10.1186/s11671-015-0770-1 PMC4385053

[56] Zanotto A, Saladino ML, Martino DC, Caponetti E 2012 Influence of temperature on calcium hydroxyapatite nanopowders. Adv. Nanoparticles 1, 21–28. (doi:10.4236/anp.2012.13004)

[57] Guha A, Sinha A 2011 Surface mineralization of hydrogels through octacalcium phosphate. Int. J. Appl. Ceram. Technol. 8, 540–546. (doi:10.1111/j.1744-7402.2010.02543.x)

[58] Combes C, Rey C 2010 Amorphous calcium phosphates: synthesis, properties and uses in biomaterials. Acta Biomater. 6, 3362–3378. (doi:10.1016/j.actbio.2010.02.017) 20167295 10.1016/j.actbio.2010.02.017

[59] Yang Z, Jiang Y, Yu L xin, Wen B, Li F, Sun S, Hou T 2005 Preparation and characterization of magnesium doped hydroxyapatite–gelatin nanocomposite. J. Mater. Chem. 15, 1807–1811. (doi:10.1039/b418015c)

[60] Devanand Venkatasubbu G, Ramasamy S, Ramakrishnan V, Kumar J 2011 Hydroxyapatite-alginate nanocomposite as drug delivery matrix for sustained release of ciprofloxacin. J. Biomed. Nanotechnol. 7, 759–767. (doi:10.1166/jbn.2011.1350) 22416574 10.1166/jbn.2011.1350

[61] Chen F, Li C, Zhu Y-J, Zhao X-Y, Lu B-Q, Wu J 2013 Magnetic nanocomposite of hydroxyapatite ultrathin nanosheets/Fe3O4 nanoparticles: microwave-assisted rapid synthesis and application in pH-responsive drug release. Biomater. Sci. 1, 1074 (doi:10.1039/c3bm60086f) 10.1039/c3bm60086f 32481873

[62] Gu L, He X, Wu Z 2014 Mesoporous hydroxyapatite: preparation, drug adsorption, and release properties. Mater. Chem. Phys. 148, 153–158. (doi:10.1016/j.matchemphys.2014.07.024)

[63] Li D, Zhu Y, Liang Z 2013 Alendronate functionalized mesoporous hydroxyapatite nanoparticles for drug delivery. Mater. Res. Bull. 48, 2201–2204. (doi:10.1016/j.materresbull.2013.02.049)

[64] Verma G, Barick KC, Manoj N, Sahu AK, Hassan PA 2013 Rod-like micelle templated synthesis of porous hydroxyapatite. Ceram. Int. 39, 8995–9002. (doi:10.1016/j.ceramint.2013.04.100)

[65] Kiurski J, Adamović S, Oros I, Krstić J, Kovačević I 2012 Adsorption feasibility in the Cr(total) ions removal from waste printing developer. Glob. Nest J. 14, 18–23.

[66] Kiurski J, Adamovic S, Krstic J 2011 Adsorption efficiency of low-cost materials in the removal of Zn (II) ions from printing developer. Acta Technica Corviniensis Bull. Eng. 4, 61–66.

[67] Manatunga DC, de Silva RM, de Silva KMN, Ratnaweera R 2016 Natural polysaccharides leading to super adsorbent hydroxyapatite nanoparticles for the removal of heavy metals and dyes from aqueous solutions. RSC Adv. 6, 105 618–105 630. (doi:10.1039/C6RA22662K)

[68] Deniz F 2013 Color removal from aqueous solutions of metal-containing dye using pine cone. Desalin. Water Treat. 51, 4573–4581. (doi:10.1080/19443994.2012.751882)

[69] Tomczak E, Tosik P 2017 Waste plant material as a potential adsorbent of a selected azo dye. Chem. Process Eng. 38, 283–294. (doi:10.1515/cpe-2017-0021)

[70] Terangpi P, Chakraborty S 2016 Adsorption kinetics and equilibrium studies for removal of acid azo dyes by aniline formaldehyde condensate. Appl. Water Sci. 7, 3661–3671. (doi:10.1007/s13201-016-0510-4)

[71] Memon S, Bhatti AA, Bhatti AA 2017 Calix[4]arene resin, an efficient adsorbent for azo dyes. Polycycl. Aromat. Compd. 5, 1–10. (doi:10.1080/10406638.2017.1306571)

[72] Cai Z, Sun Y, Liu W, Pan F, Sun P, Fu J 2017 An overview of nanomaterials applied for removing dyes from wastewater. Environ. Sci. Pollut. Res. 24, 15 882–15 904. (doi:10.1007/s11356-017-9003-8) 10.1007/s11356-017-9003-8 28477250

