# Supplementary material for: Metal and polymer-mediated synthesis of porous crystalline hydroxyapatite nanocomposites for environmental remediation
Source: R Soc Open Sci. 2018 Jan 24;5(1):171557. doi: 10.1098/rsos.171557 (PMC5792936; doi:10.1098/rsos.171557)
Supplement: Characterization data and Isotherm data [file rsos171557supp1.docx]

**Metal and polymer mediated synthesis of porous crystalline hydroxyapatite nanocomposites for environmental remediation**

Danushika. C. Manatunga,^a^ Rohini. M. de Silva^a^, Nuwan de Silva^b^, E.V.A. Premalal^b^, K. M. Nalin de Silva^a,b^

**Characterization Data and Isotherm Data**

**Supporting Infroamtion**

**1. Experimental details**

**1.1. Isotherm models used for adsorption studies**

The Langmuir model assumes the monolayer adsorption of molecules on to the surface of the adsorbate, which consists of a finite number of identical sites. Its linear form can be given by the following equation,

$$\frac{\boldsymbol{C}_{\boldsymbol{e}}}{\boldsymbol{Q}_{\boldsymbol{e}}}\boldsymbol{=}\frac{\boldsymbol{1}}{\boldsymbol{q}_{\boldsymbol{m}}\boldsymbol{K}}\boldsymbol{+}\frac{\boldsymbol{C}_{\boldsymbol{e}}}{\boldsymbol{q}_{\boldsymbol{m}}}\boldsymbol{-----(1)}$$

where C_e_ is the equilibrium concentration, Q_e_ is the equilibrium sorption capacity (mg/g), K (L/mg) is the equilibrium adsorption constant which correlates with the affinity of the binding sites and q_m_ (mg/g) is the maximum amount of dye adsorbed on to a unit mass of the adsorbent when all the binding sites are being occupied by the adsorbate.

The Freundlich isotherm is the other model which was used to describe the isotherm patterns. This hypothesizes the adsorption on heterogeneous surfaces through a multilayer adsorption process. The empirical formula of the Freundlich isotherm model can be expressed as follows,

$$\ln\boldsymbol{Q}_{\boldsymbol{e}}\boldsymbol{=}\ln\boldsymbol{K}_{\boldsymbol{f}}\boldsymbol{+}\frac{\boldsymbol{1}}{\boldsymbol{n}}\ln\boldsymbol{C}_{\boldsymbol{e}}\boldsymbol{----}\left( \boldsymbol{2} \right)$$

where K_f_ (mg/g(mg/L)^-1/n^) and n denote the Freundlich constants that are related to the adsorption capacity and the adsorption intensity of the adsorbate molecules. These two isotherm models were used to understand the mechanism of adsorption of solutes from the aqueous solution.

**1.2. Effect of the contact time**

For this purpose, eight separate containers containing 0.02 g of adsorbent were prepared, 10.0 mL of 200 ppm Acid Yellow dye solution were added to each, and mixtures were agitated at 200 rpm at room temperature, at pH 6 for different time intervals, as given in the supplementary data. The absorbance was recorded at wavelength (λ_max_) of 436 nm for each system in given time intervals. The residual dye amount remaining in the solution was analysed to find the sorption capacity as follows,

$$Q_{e}=\frac{\left( C_{0}-C_{t} \right)V}{m}-----\left( 3 \right)$$

Where *Q_e_* is the equilibrium sorption capacity (mg/g), *C_o_* and *C_t_* are the concentration in solution before and after the adsorption (mg/dm^3^) respectively, *m* is the amount of matter in the reaction mixture (g), and *V* is the volume of the medium (dm^3^).

**1.3. Effect of pH on dye removal**

The effect of pH for the adsorption process was also investigated. Briefly, 0.010 g from each adsorbent was added to a dye solution having a concentration of 200 ppm, and it was agitated at 200 rpm over the pH range of 2.5 - 7.5 for a specific period of time (the experimentally determined optimum time). The residual dye amount remaining in the solution was collected by centrifugation and analysed spectrophotometrically to find the sorption capacity as given in Eq. (3).

**1.4. Batch sorption experiments of the dye**

An aliquot of 0.025 g of each adsorbent was added to 10.0 mL of the freshly prepared dye solution of concentration 50, 100, 200, 300, 400, and 500 ppm, respectively. Each mixture was then agitated at 200 rpm for the experimentally determined optimum time of each adsorbent at room temperature. The aqueous dye layer was then separated using centrifugation and the concentration was determined using the UV/Visible spectrophotometer. The data obtained was fitted into the two isotherm models, Freundlich and Langmuir isotherms.

**2. Results**

**
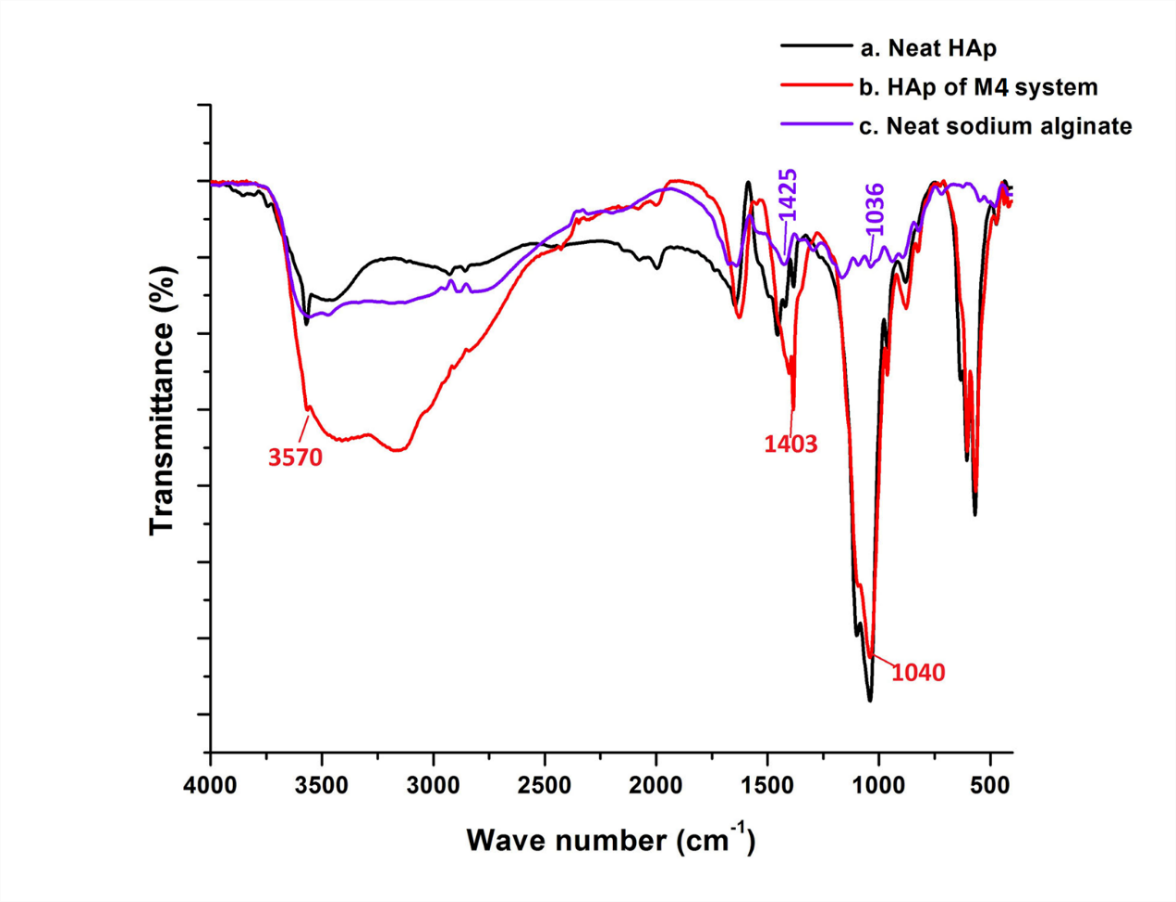
**

Figure. S1. FT-IR spectrum comparison of M4 system with neat HAp and neat NaAlg polymer

**
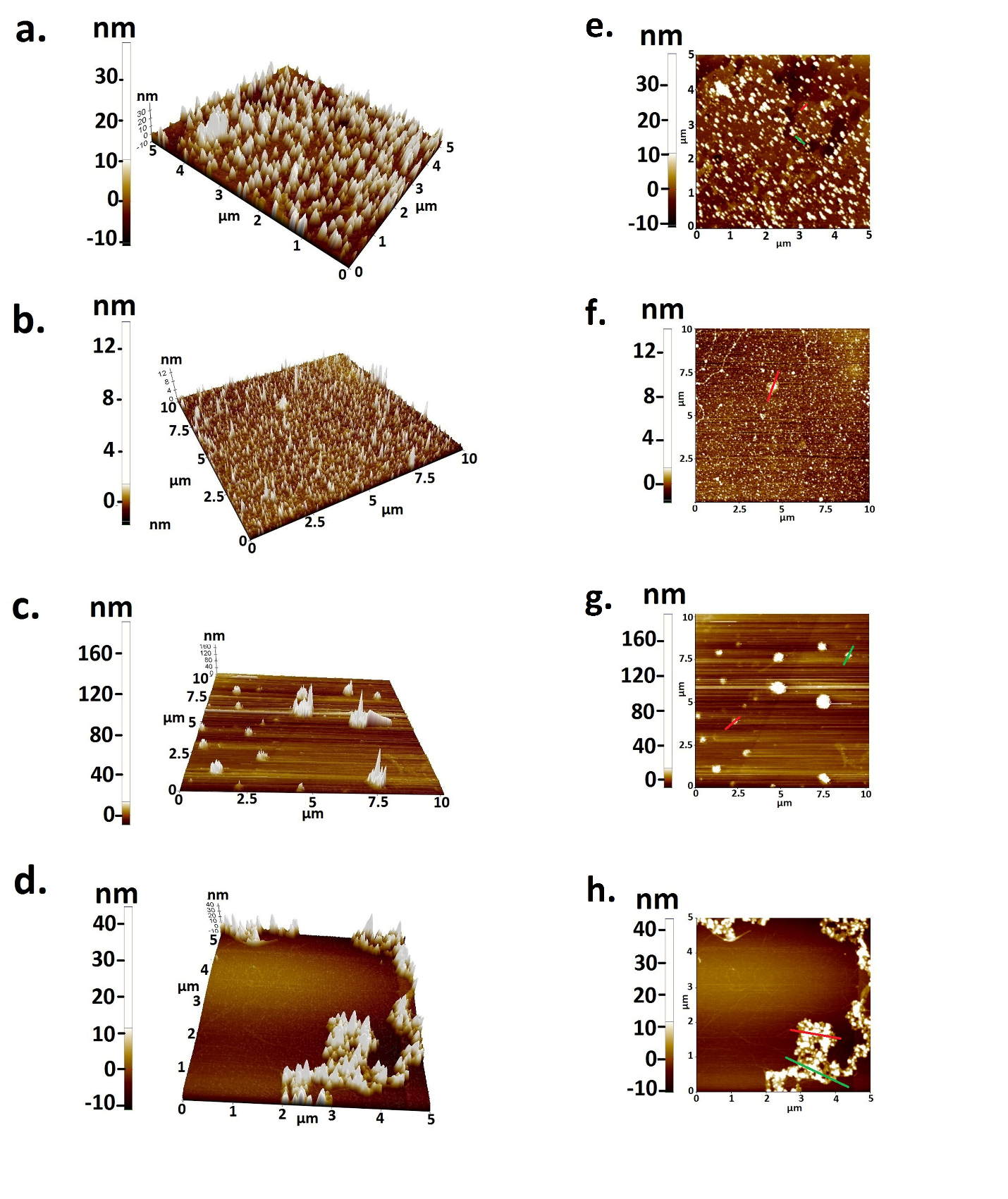
**

Figure. S2. AFM images of synthesized HAp nanoparticles via different approaches. (a-d, 3D topographic image of M1-M4 and e-h, 2D topographic image of M1-M4)

Table S1. Line width of ^1^HNMR peak at 5.3-5.5 ppm of each system

| System | Line width (ppm) |
| --- | --- |
| Neat HAp | 3.0105 |
| M1 | 0.1934 |
| M2 | 3.5391 |
| M3 | 7.1968 |
| M4 | 1.9569 |

Table S2. Peak position and Line width of ^31^PNMR peak at 3.2 ppm of each system

| System | Peak position (ppm) | Line width (ppm) |
| --- | --- | --- |
| Neat HAp | 3.2852 | 0.7945 |
| M1 | 3.2860 | 0.9289 |
| M2 | 3.3822 | 5.4288 |
| M3 | 3.3211 | 6.0777 |
| M4 | 3.2548 | 1.7274 |

Table S3. Chemical composition of hydroxyapatite by XRF studies

| System | Ca/P molar ratio or (Ca+dopant)/P molar ratio |
| --- | --- |
| M1 | 0.84 |
| M2 | 0.53 |
| M3 | 0.66 |
| M4 | 1.57 |


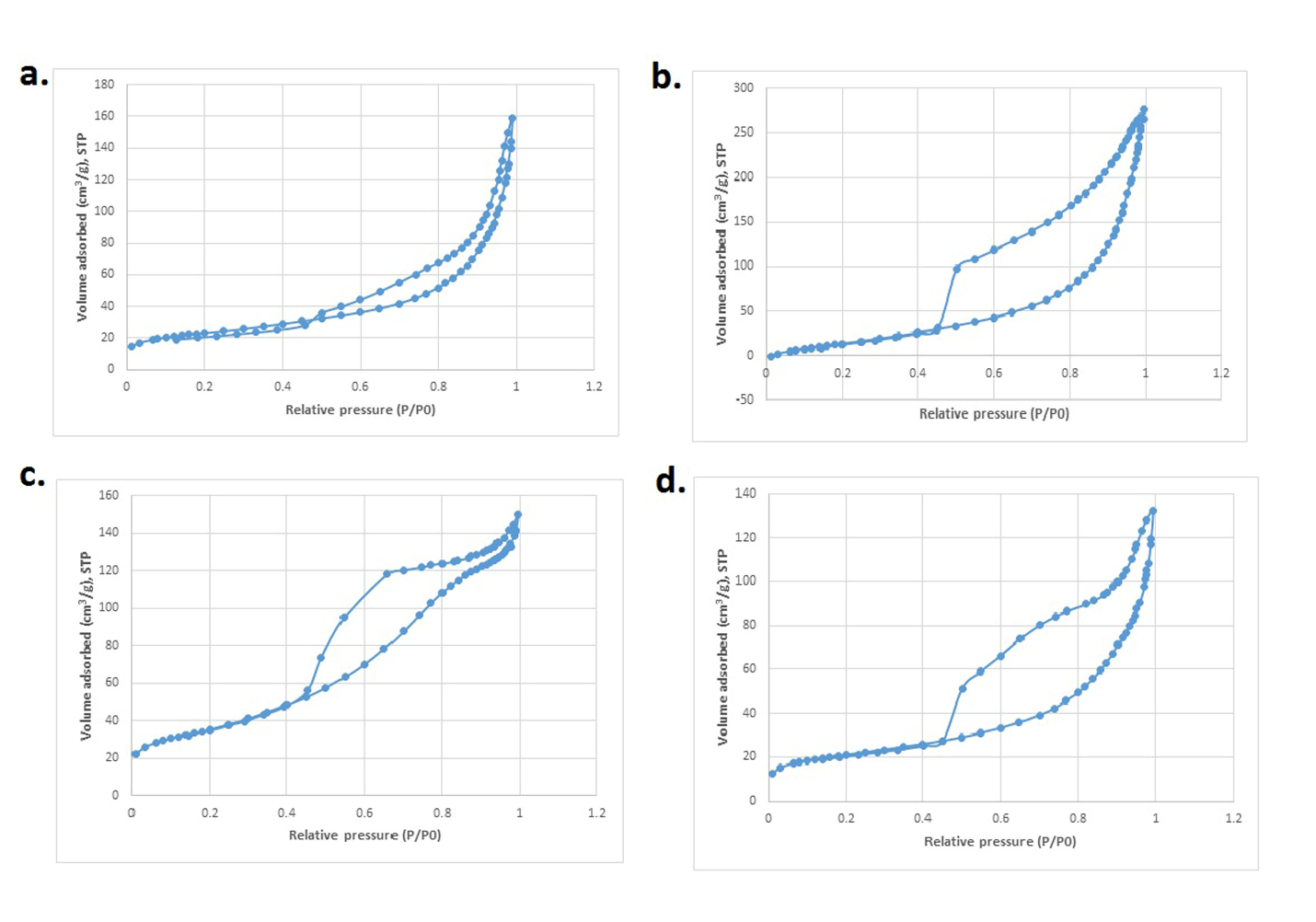


Figure. S3. N_2_ adsorption/desorption isotherms for a) M1, b) M2, c) M3 and d) M4

Table S4. Surface characteristics of the prepared HAp nanoparticulate systems

| Sample | S_BET_(m^2^/g) | Average pore diameter (nm) | BJH cumulative desorption pore volume (cm^3^/g) |
| --- | --- | --- | --- |
| M1 | 82.22 | 10.55 | 0.2340 |
| M2 | 81.82 | 19.27 | 0.4155 |
| M3 | 126.5 | 6.780 | 0.2283 |
| M4 | 74.49 | 9.718 | 0.1999 |


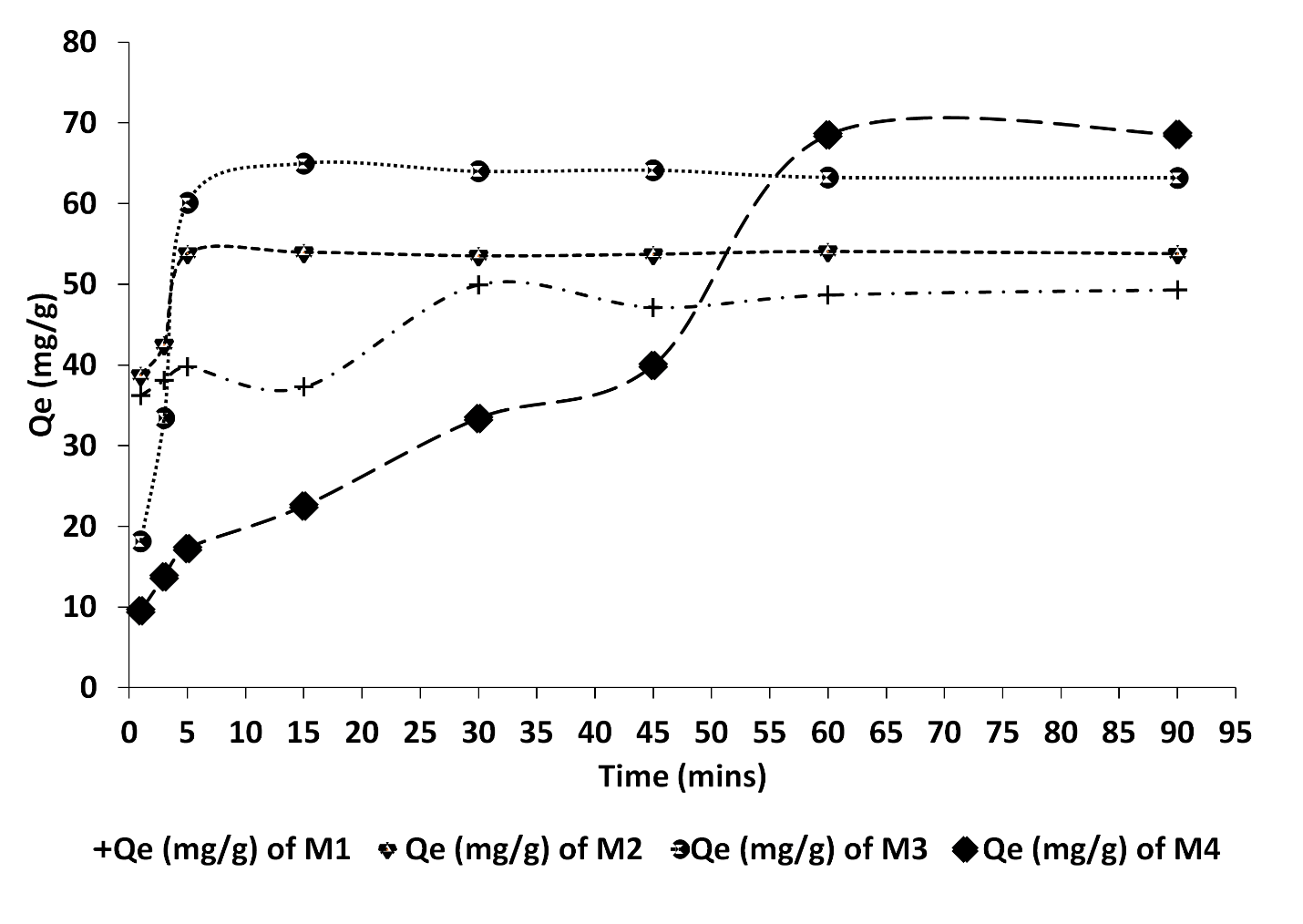


Figure. S4. Effect of contact time on the equilibrium adsorption of Acid yellow 220 by different HAp systems when they were incubated with 200 ppm solution of acid yellow 220.

Table S5. Equilibrium percentage removal of Acid yellow 220 by each apatite system

| System | Equilibrium percentage removal (%) |
| --- | --- |
| M1 | 24.97 |
| M2 | 26.93 |
| M3 | 32.49 |
| M4 | 65.72 |


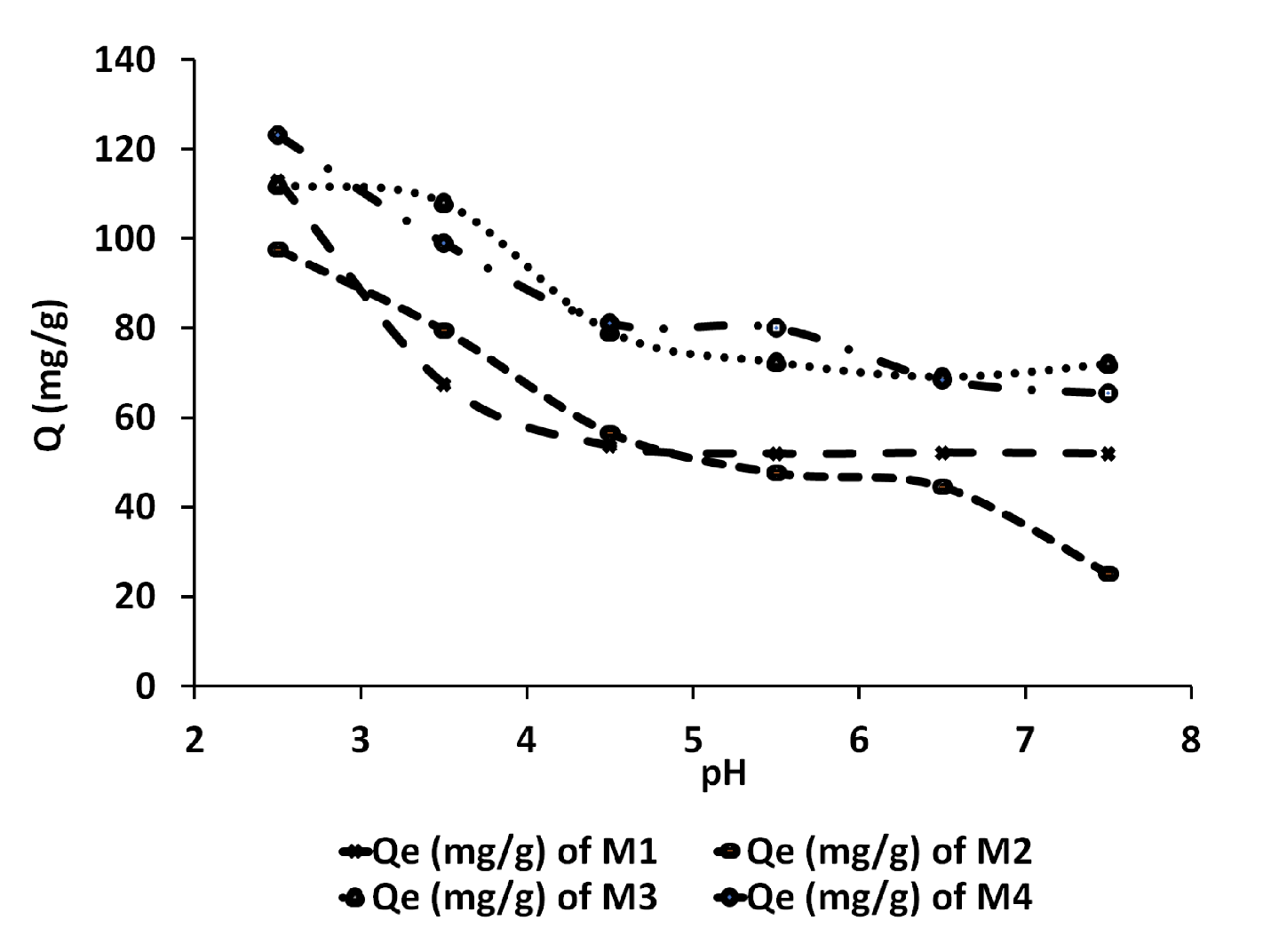


Figure. S5. Effect of pH variation on the equilibrium adsorption of Acid yellow 220 by different HAp systems


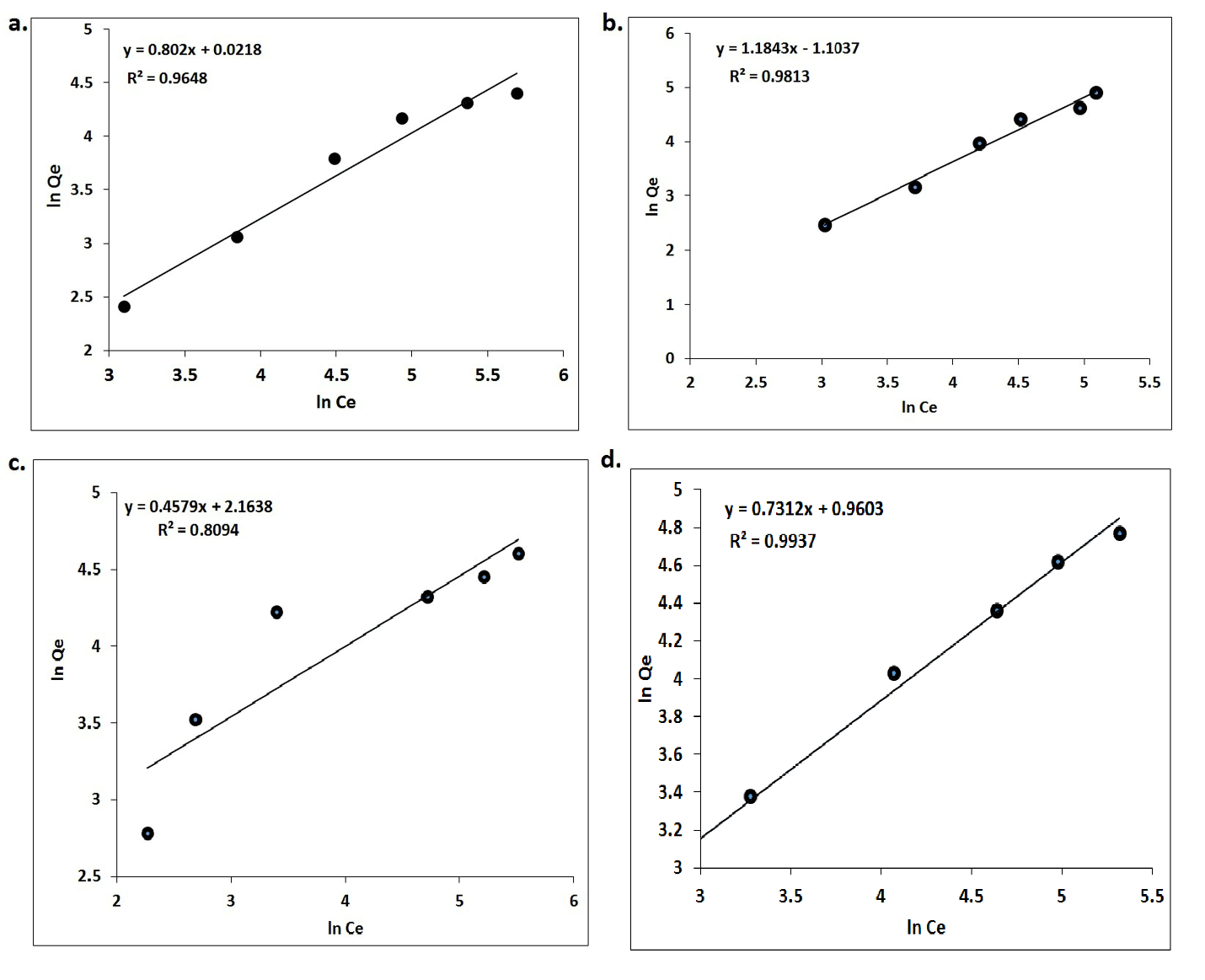


Figure. S6. Freundlich isotherm pattern of each system over the adsorption of Acid yellow 220 a) M1, b) M2, c) M3 and d.) M4


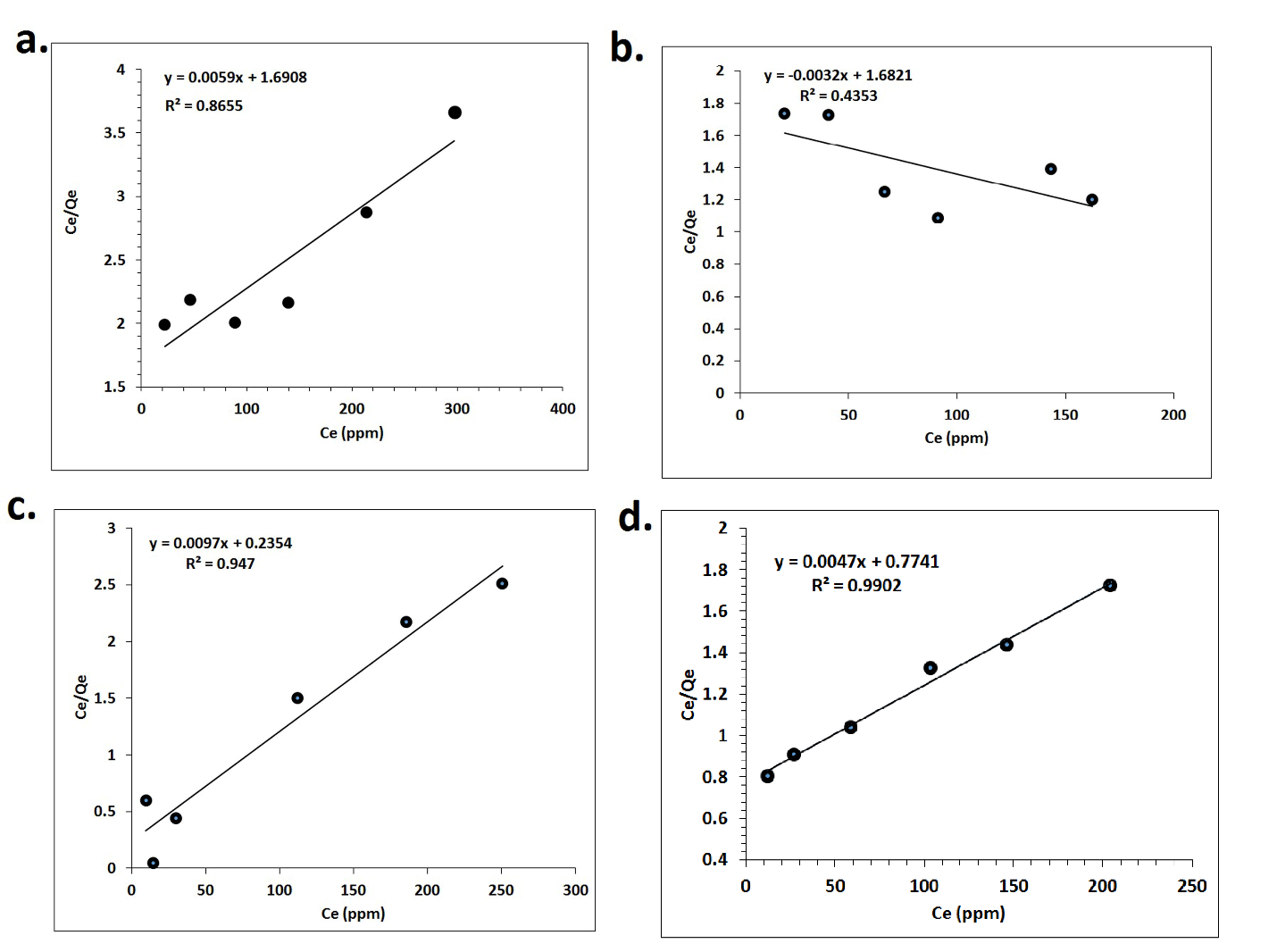


Figure. S7. Langmuir isotherm pattern of each system over the adsorption of Acid yellow 220 a) M1, b) M2, c) M3 and d.) M4


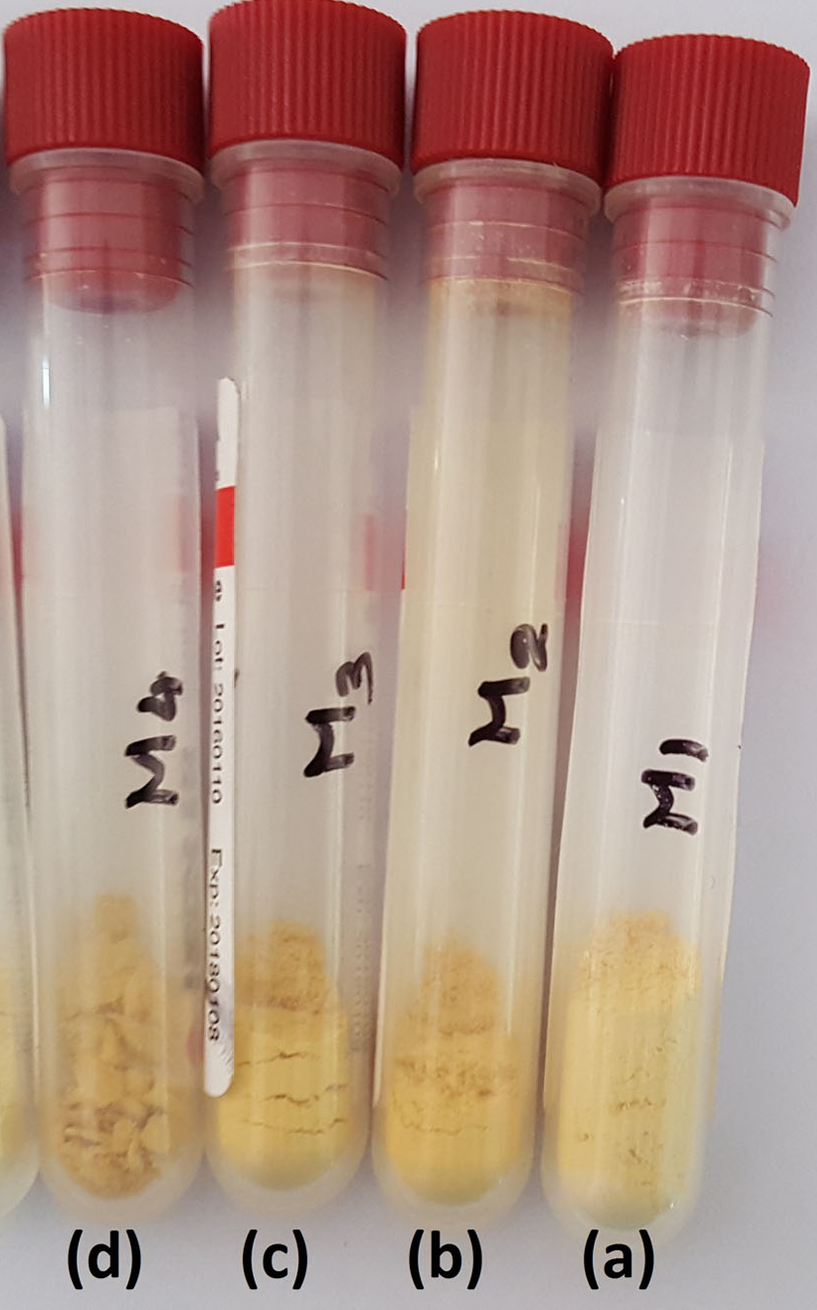


Figure. S8. Color of HAp nanoparticles after Acid yellow 220 dye binding a) M1, b) M2, c) M3 and d) M4


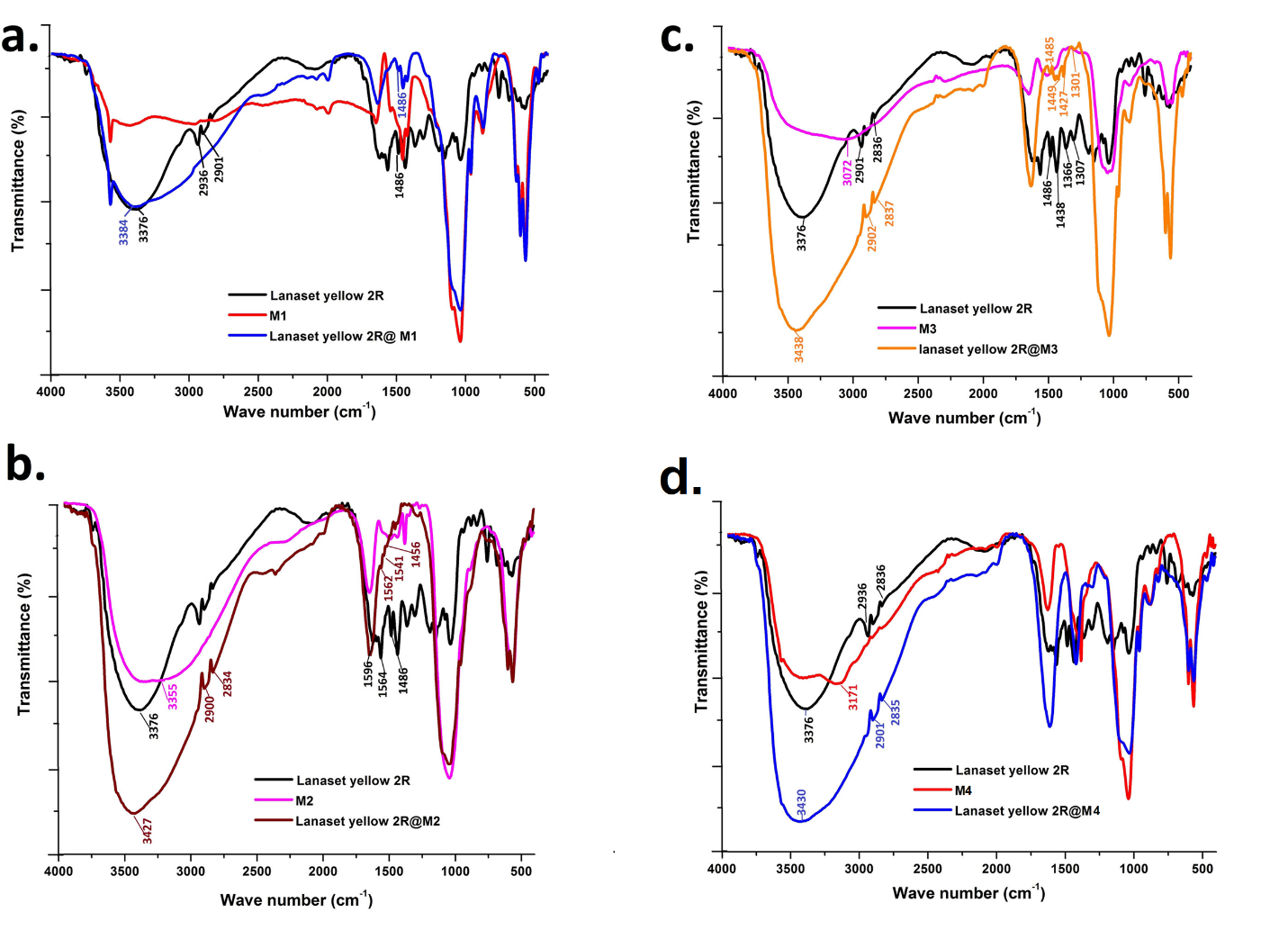


Figure. S9. FT-IR characterization of dye bound HAp nanoparticles (a-d: M1-M4 systems)

When the Lanaset 2R interacts with the M1 HAp system, it is clearly seen that the broadness of –OH of the dye bound HAp has increased while having a shift from 3376 cm^-1^ (neat dye) to 3384 cm^-1^(dye bound HAp) [^[[1]](#footnote-1)^] And the –OH bands might have shifted to higher frequencies after the dye adsorption **[**^[[2]](#footnote-2)^**].** The appearance of the –CH_2_ symmetric and asymmetric stretching vibrations of the dye [1,^[[3]](#footnote-3)^] were not clearly visible as in the case of neat dye. Peak at 1486 cm^-1^ which could corresponds to the stretch of C=C on the benzene ring [^[[4]](#footnote-4)^] was appearing both in the neat dye and dye bound M1 which confirmed the binding of the dye molecules to the M1 HAp surface. But due to the absence of strong absorption bands corresponding to the dye molecules and the appearance of more of hydroxyapatite absorption bands indicate that the amount of dye molecules lying on the HAp surface could be a little and more of them might have entrapped inside the HAp nanoparticles.

In M2 HAp system, after the dye binding the –OH stretching vibrational band has become intense with respect to the neat M2 HAp. This could be possibly due to the interaction of free –OH groups of the HAp with the dye molecules after the binding.^2^ Moreover, the CH_2_ stretching vibrations of the dye molecules^1,3,^^[[5]](#footnote-5)^ are clearly seen in the dye bound M2 HAp system. This confirms more of the dye molecules are residing on the HAp surface and they are free to vibrate with respect to the dye bound M1 system. The aromatic CH bending and CH in plane stretching vibrational bands appearing in the dye^[[6]](#footnote-6)^ at 1596 cm^-1^, 1564 cm^-1^ and 1486 cm^-1^ have shifted to lower wave numbers with the binding to the M2 HAp surface (1562 cm^-1^, 1541 cm^-1^ and 1456 cm^-1^).

In the systems M3 also after dye binding the -CH_2_ stretching vibrations and the dye vibrational bands at 1486 cm^-1^, 1438 cm^-1^, 1366 cm^-1^ and 1301 cm^-1^ are clearly seen with a shift to the higher wave numbers in the dye bound M3 complex. Moreover, the –OH vibrational band has become intense after the dye binding as similar to M2.

In the system M4, when the dye is attached to HAp, there is the appearance of -CH_2_ stretching vibrational bands as similar to other systems (M2, M3 and M4). Here also the -OH stretching vibrational band broadness and the intensity have significantly increased. The appearance of the vibrational bands of the dye bound HAp is very similar to M2 and M3. Other than that the peaks corresponding to HAp of the dye bound HAp, have given rise to a reduced intensity (increased broadness) when compared with the neat HAp (M4) and this could be due to the interaction of HAp with the dye molecules.

1. R. K. Gautam, S. Banerjee, P.K. Gatam, V. Rawat, A. Kumar. A, S. K. Singh, M. C. Chattopadhyaya, Biosorption of an Acidic Dye, Alizarin Red S, Onto Biosorbent of Mustard husk: Kinetic, Equilibrium Modeling and Spectroscopic Analysis, Asian J. Res. Chem 7(2014) 417–425. [↑](#footnote-ref-1)
2. D. Sun, Z. Zhang, M. Wang, Y. Wu, Adsorption of Reactive Dyes on Activated Carbon Developed from Enteromorpha prolifera Deshuai, Am. J. Anal. Chem 4 (2013) 17–26. [↑](#footnote-ref-2)
3. O. Koçer, B. Acemioğlu, Adsorption of Basic green 4 from aqueous solution by olive pomace and commercial activated carbon: process design, isotherm, kinetic and thermodynamic studies Desalin. Water Treat 57 (2016) 16653–16669. [↑](#footnote-ref-3)
4. H. Zhang, C. Ding, J. Cao, W. Xu, X. Li, H. Zhang, A novel solvent-template method to manufacture nano-scale porous membranes for vanadium flow battery applications, J. Mater. Chem. A, 2 (2014) 9524. [↑](#footnote-ref-4)
5. B. T. Pelosi, L. K.S. Lima, M. G. A. Vieira, REMOVAL OF THE SYNTHETIC DYE REMAZOL BRILLIANT BLUE R FROM TEXTILE INDUSTRY WASTEWATERS BY BIOSORPTION ON THE MACROPHYTE Salvinia natans, Brazilian J. Chem. Eng., 31 (2014) 1035–1045. [↑](#footnote-ref-5)
6. T. B. V. Neves, G. F. S. Andrade, SERS Characterization of the Indocyanine-Type Dye IR-820 on Gold and Silver Nanoparticles in the Near Infrared. *J. Spectrosc* 2015, (2015) 1-9. [↑](#footnote-ref-6)
